# More Gamma More Predictions: Gamma-Synchronization as a Key Mechanism for Efficient Integration of Classical Receptive Field Inputs with Surround Predictions

**DOI:** 10.3389/fnsys.2016.00035

**Published:** 2016-04-25

**Authors:** Martin Vinck, Conrado A. Bosman

**Affiliations:** ^1^School of Medicine, Yale UniversityNew Haven, CT, USA; ^2^Cognitive and Systems Neuroscience Group, Swammerdam Institute, Center for Neuroscience, University of AmsterdamAmsterdam, Netherlands; ^3^Facultad de Ciencias de la Salud, Universidad Autónoma de ChileSantiago, Chile

**Keywords:** gamma oscilations, predictive coding, efficient coding, surround suppression, V1, gamma synchrony, communication through coherence, laminar organization

## Abstract

During visual stimulation, neurons in visual cortex often exhibit rhythmic and synchronous firing in the gamma-frequency (30–90 Hz) band. Whether this phenomenon plays a functional role during visual processing is not fully clear and remains heavily debated. In this article, we explore the function of gamma-synchronization in the context of predictive and efficient coding theories. These theories hold that sensory neurons utilize the statistical regularities in the natural world in order to improve the efficiency of the neural code, and to optimize the inference of the stimulus causes of the sensory data. In visual cortex, this relies on the integration of classical receptive field (CRF) data with predictions from the surround. Here we outline two main hypotheses about gamma-synchronization in visual cortex. First, we hypothesize that the precision of gamma-synchronization reflects the extent to which CRF data can be accurately predicted by the surround. Second, we hypothesize that different cortical columns synchronize to the extent that they accurately predict each other’s CRF visual input. We argue that these two hypotheses can account for a large number of empirical observations made on the stimulus dependencies of gamma-synchronization. Furthermore, we show that they are consistent with the known laminar dependencies of gamma-synchronization and the spatial profile of intercolumnar gamma-synchronization, as well as the dependence of gamma-synchronization on experience and development. Based on our two main hypotheses, we outline two additional hypotheses. First, we hypothesize that the precision of gamma-synchronization shows, in general, a negative dependence on RF size. In support, we review evidence showing that gamma-synchronization decreases in strength along the visual hierarchy, and tends to be more prominent in species with small V1 RFs. Second, we hypothesize that gamma-synchronized network dynamics facilitate the emergence of spiking output that is particularly information-rich and sparse.

## Introduction

The natural world contains statistical regularities that create correlations in the input across sensory channels. The brain can use these regularities to improve the efficiency of neural representations, and optimize stimulus inference (Rao and Ballard, 1999; Simoncelli and Olshausen, 2001; Weiss et al., 2002; Doya et al., 2011; Bastos et al., 2012; Sachdev et al., 2012). This principle forms the foundation of theories of efficient and predictive coding, and has been used to explain numerous response properties of cells in the visual system. These include the high-pass filtering properties of cells in the retina and the lateral geniculate nucleus (LGN), as well as the existence of sparse receptive fields (RFs) in area V1 (Olshausen and Field, 1996; Simoncelli and Olshausen, 2001; Doi et al., 2012). There is also evidence that contextual modulations of V1 firing rates, such as end-stopping and surround suppression, reflect the integration of local LGN input with predictions from the surround (Rao and Ballard, 1999; Vinje and Gallant, 2000; Spratling, 2010; Pecka et al., 2014; Coen-Cagli et al., 2015). Here we explore the cortical dynamics supporting the integration of local, bottom-up inputs with predictions from the surround, an integration that involves large-scale, recurrent interactions between distributed information sources.

**Figure 1:**
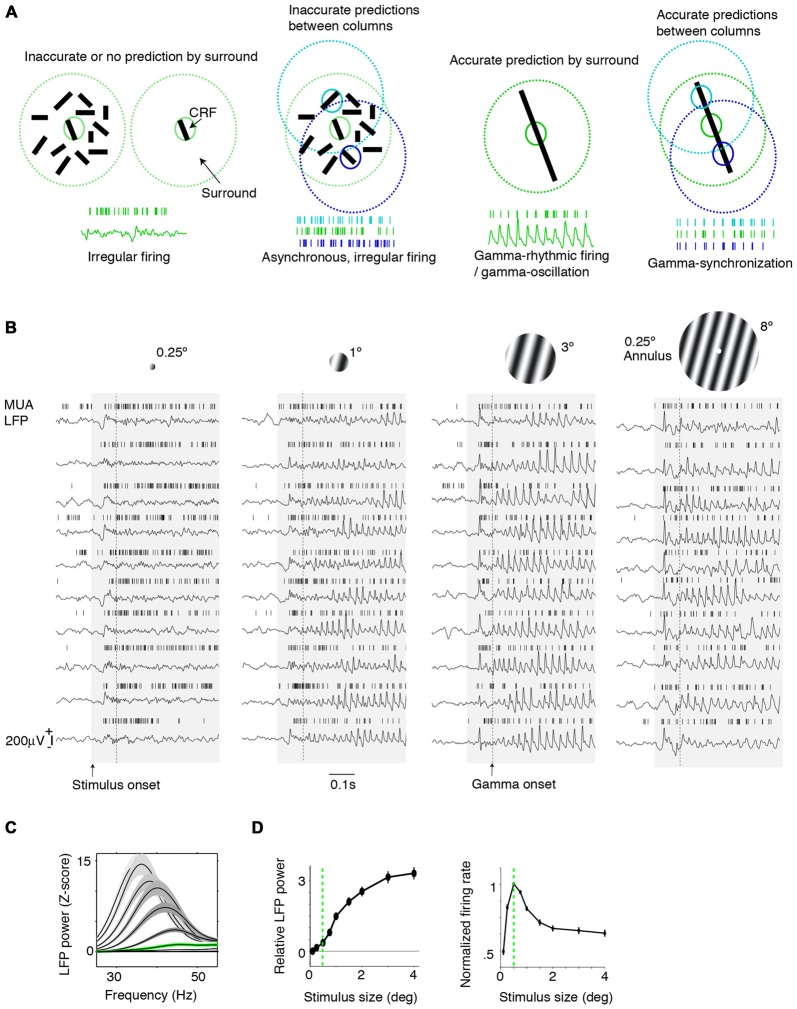
**Dependence of gamma-synchronization on stimuli properties. (A)** Schematic overview of *Principles 1* and *2* (see “Introduction” and “The Relationship Between Gamma-Synchronization and Geometry” Sections). Gamma-synchronization emerges when there is a predictive relationship between surround and classical receptive field (CRF) data. Neurons fire irregularly when the CRF content is not accurately predicted by the surround (*Principle 1*). Different columns gamma-synchronize if they predict each other’s visual input accurately, but fire asynchronously otherwise (*Principle 2*). See Chen et al. (2014) for the lack of gamma-synchronization with an array of randomly oriented lines. **(B)** Gieselmann and Thiele (2008) performed V1 recordings from awake monkeys which were passively viewing stationary gratings. Shown are multiple trials with LFP and multi-unit traces recorded from the same electrode. Gray shading indicates stimulus onset. Strong gamma-synchronization is observed for gratings larger than the CRF, but gamma-synchronization is not detected for a small stimulus. For large gratings, spikes exhibit phase locking to LFP. Dashed line indicates approximate onset of first induced gamma peaks/troughs around 100 ms. Surround suppression already occurs before the onset of gamma (Xing et al., 2005; Gieselmann and Thiele, 2008). In the 0.15–0.5 s period, firing variability is high for the small 0.25° stimulus (Fano Factor 3.18) while it is low for the large 3° stimulus (Fano Factor 0.81). Right: monkeys viewed a large grating stimulus of 8° with a small modification of CRF structure (annulus mask) that diminishes the accuracy of surround predictions. This stimulus is accompanied by reduced gamma-synchronization compared to the case of a large coherent grating. For this annulus stimulus, the average reduction in 20–100 Hz gamma-power is about 50% compared to the case of a 4° grating, such that gamma-synchronization is at the same level as a grating of about 1.25° (Gieselmann and Thiele, 2008). Assuming that the annulus is precisely centered around the recorded neuron’s CRF, it should cover an area of about 25% of the CRF. If the annulus is not precisely centered around the recorded neuron’s CRF, it will cover a smaller area. **(C)** Average *z*-scored LFP power spectra relative to baseline. In green, the curve for the median CRF size (0.5°). **(D)** Change in gamma power (20–100 Hz; left) and firing rate (right) with size. **(B–D)** Adapted from Gieselmann and Thiele (2008).

The canonical picture of neural firing in sensory cortex is that it is highly irregular and exhibits a large variability of inter-spike-intervals. Yet, during visual stimulation, cortical dynamics in visual cortex are often characterized by rhythmic and synchronous firing in the gamma-frequency band (Gray et al., 1989; 30–80 Hz; Figure 1). There is a rich history of work examining this phenomenon. The functional significance of empirical findings on gamma-synchronization were initially explored in the context of the so-called “binding problem” (Engel et al., 1991, 1992; Singer and Gray, 1995). This refers to the problem that the brain segments images into segregated objects, which necessitates that the local features comprising the object must at some processing stage be dynamically bound together or “tagged”. It has been proposed that the activity of distributed neurons responding to the same object can be dynamically grouped together through synchrony (Milner, 1974; Engel et al., 1992; Singer and Gray, 1995; Gray, 1999; Shadlen and Movshon, 1999; Singer, 1999; von der Malsburg, 1999; BBS – “binding by synchronization”). However, the object-specific synchrony predicted by BBS was found by some, but not by all studies (Roelfsema et al., 2004; Palanca and DeAngelis, 2005; Ramalingam et al., 2013). Moreover, it remains unclear how to reconcile the many stimulus-dependencies of gamma-synchronization (e.g., on size, texture and motion) with the idea of object-specific synchrony (Gieselmann and Thiele, 2008; Zhou et al., 2008).

Later studies on gamma-synchronization placed a strong emphasis on its functional role in regulating information flow between brain areas (Kopell et al., 2000; Salinas and Sejnowski, 2001; Varela et al., 2001; Tiesinga et al., 2004; Fries, 2005; Sejnowski and Paulsen, 2006; Fries et al., 2007; Börgers and Kopell, 2008; Lowet et al., 2015; Bastos et al., 2015b). For example, Fries (2005, 2015) proposed that communication between neuronal groups can be flexibly modulated through coherence as a function of cognitive demands, such as attention, and several groups have now shown that spatial attention can indeed selectively modulate gamma-coherence between visual areas (Gregoriou et al., 2009; Bosman et al., 2012; Grothe et al., 2012). Yet, it has been argued that the purported role of gamma-coherence in selective attention is at odds with the strong dependence of gamma on bottom-up stimulus characteristics (Jia et al., 2013a; Ray and Maunsell, 2015). Thus, despite major conceptual advances provided by these theories, the role of gamma-synchronization during visual perception has not been fully unveiled yet.

### Main Hypotheses

In this article, we hypothesize that the stimulus dependencies of gamma-synchronization can be explained using the framework of predictive and efficient coding. It is known that in primary visual cortex, each cortical column receives a direct projection from the thalamus (which in turn receives/relays information from the retina), which carries information about only a small portion of the visual field (the classical receptive field, CRF). In addition, the spiking activity of its constituent neurons can, through lateral and extrastriate feedback connections, be modulated by information outside the CRF (which is commonly referred to as the “surround”; Gilbert, 1992; Lund et al., 2003). We hypothesize that the visual cortex operates under two different coding strategies and utilizes each depending on the structure of the visual input. At one extreme, consider a scenario where the input data to cortical columns having non-overlapping CRFs is largely independent, e.g., for a white noise texture. In this case, spiking output is irregular, and cells in different columns fire asynchronously (Figures 1A,B). At the opposite extreme, when the surround data reliably predict the CRF data, superficial layers use the gamma rhythm to encode information collectively. In this gamma-coordinated coding regime, cells send and receive information synchronously in 30–80 Hz cycles (Figures 1A,B). In effect, the coding mode resides on a continuum between these two extremes and we propose the following principles to describe this:

*Principle 1*: The strength of gamma-synchronization reflects the extent to which the visual input in the CRF can be accurately predicted from the surround.

*Principle 2*: Populations with non-overlapping CRFs will gamma-synchronize to the degree that they accurately predict each other’s visual input (Figure 1A).

Note that these two principles separately assess two different phenomena, namely, the strength of gamma-synchronization between the neurons in a local column and the gamma-rhythmicity of those neurons (first principle), and the gamma-synchronization of neurons with non-overlapping CRFs (second principle).

Based on these two principles, we propose two additional hypotheses concerning gamma-synchronization and its stimulus dependencies. First, we hypothesize that the precision of gamma-synchronization shows, in general, a negative dependence on RF size. Second, we hypothesize that gamma-synchronized network dynamics facilitate the integration of surround with CRF data, resulting in stimulus representations that are both more sparse and information-rich (see “Relationship Between Gamma-Synchronization and Firing Rate Coding” and “Functional Consequences of Gamma-Synchronization” Section).

### Outline

The article is organized as follows. In the first part of this article, we will review empirical studies supporting our main hypothesis and the two proposed principles (see “The Relationship Between Gamma-Synchronization and Geometry”, “The Relationship Between Gamma-Synchronization and Motion Properties” and “Stimulus-Dependence of Intercolumnar Gamma-Synchronization” Sections). Next, we discuss the relationship between the stimulus correlates of gamma and its circuit mechanisms (see “Mechanisms of Gamma-Synchronization” Section). In “Gamma-Synchronization Depends on Experience and Development” Section, we consider how gamma-synchronization is shaped by learning the natural statistics of the environment through development and experience. We then examine how the outlined principles and mechanisms apply to higher visual areas and different species, and hypothesize that CRF size is a key determinant of gamma-synchronization strength (see “Gamma Across the Visual Hierarchy” Section). We proceed by exploring the functional role that gamma-synchronization plays in the encoding and transmission of information (see “Relationship Between Gamma-Synchronization and Firing Rate Coding” and “Functional Consequences of Gamma-Synchronization” Sections).

### Preliminaries

(i) We initially focus this review on area V1 (striate cortex), because, compared to extrastriate cortex, the properties of V1 gamma have been studied more extensively and V1 gamma-synchronization tends to be stronger (see “Gamma Across the Visual Hierarchy” Section). The application of the proposed principles to higher visual areas will be addressed in “Gamma Across the Visual Hierarchy” Section.

(ii) Because historically, many studies on gamma-synchronization have been performed in anesthetized animals, we will discuss data both from anesthetized and awake animals. It should be noted that for moving stimuli, prominent gamma-synchronization has been observed both under awake and anesthetized conditions, although it tends to be stronger in the awake condition (Gray and Viana Di Prisco, 1997). It is also known that stationary stimuli can induce robust V1 gamma-synchronization in awake primates (Gieselmann and Thiele, 2008). Yet, in anesthetized cats, V1 gamma-synchronization for stationary stimuli is much weaker than for moving stimuli (Gray et al., 1990). For this reason, we do not support our main conclusions with studies using stationary stimuli in anesthetized animals. In general, one needs to exert extreme caution with the interpretation of data from anesthetized animals: Anesthesia reduces the contextual modulations of firing rates (Lamme et al., 1998), can strongly reduce the firing of some GABAergic interneurons (Adesnik et al., 2012; Haider et al., 2013), affects signaling on the apical dendrite which is a main target of corticocortical feedback (Potez and Larkum, 2008), and promotes the occurrence of slow oscillations (UP and DOWN states) in neocortex (Steriade et al., 1993).

(iii) It is also important to consider that, like sleep and anesthesia, wakefulness is not a singular state, but consists of a rich spectrum of sub-states (Harris and Thiele, 2011; McCormick et al., 2015; McGinley et al., 2015b). In general, activation of the ascending arousal system promotes cortical desynchronization at low frequencies, as well as the emergence of V1 gamma-synchronization (Munk et al., 1996; Herculano-Houzel et al., 1999; Goard and Dan, 2009; Niell and Stryker, 2010; Harris and Thiele, 2011; Pinto et al., 2013; Lee et al., 2014; McGinley et al., 2015a; Vinck et al., 2015), with preservation of the stimulus-specificity of gamma-synchronization (Munk et al., 1996). However, because precise methods to identify behavioral states in awake animals like pupil diameter have been rarely applied to animal models (Reimer et al., 2014; McGinley et al., 2015a,b; Vinck et al., 2015), we will treat the wake state as a singular state for the purpose of this review.

(iv) It is well established that spatial attention generally leads to increments in stimulus-driven firing rates but can also modulate gamma-synchronization, although the latter effect is small in comparison to the modulation by bottom-up stimulus properties, especially in area V1 (Fries et al., 2001, 2008; Bichot et al., 2005; Taylor et al., 2005; Gregoriou et al., 2009; Chalk et al., 2010; Buffalo et al., 2011; Vinck et al., 2013a; van Kerkoerle et al., 2014). However, in this article, we are primarily concerned with the neuronal representation rather than the selection of visual information.

## Gamma-Synchronization and Stimulus Geometry

We first review evidence showing that *Principle 1* captures the many dependencies of gamma on the geometric properties of visual stimuli. When we present a stimulus input to area V1 that only covers its CRF, V1 spiking tends to be highly irregular, despite the fact that neurons fire vigorously (Figure 1A). This irregular firing pattern, which is characterized by a large variability of the inter-spike-intervals, is the classic picture of neuronal output that is the cornerstone of many computational network models. Yet, a radically different picture emerges when we present a large stimulus that covers both the CRF and the surround of V1 neurons. If the stimulus allows for accurate predictions of a neuron’s CRF input from its surround, e.g., in case of a regular texture (grating or checkerboard) or a bar stimulus, then its spiking output tends to become remarkably rhythmic (Gray et al., 1989; Gieselmann and Thiele, 2008; Figures 1A–D). This rhythmicity is shared by a large fraction of cells in the local column, resulting in a gamma-synchronous pattern of network activity, with spectral energy focused in the 30–80 Hz frequency band (Gray et al., 1989, 1990; Livingstone, 1996; Maldonado et al., 2000). While some minimum level of gamma-synchronization may exist for stimuli that are smaller than the CRF or for baseline conditions without visual stimulation, especially in fast spiking (FS) interneurons (Vinck et al., 2013a; Lewis et al., 2016; Perrenoud et al., 2016), it is apparent that a narrow frequency-band emerges only once stimuli extend beyond the CRF border (Figures 1B–D). We further note that the strong gamma-synchronization observed for large, regular textures occurs even though neurons fire at lower rates than those observed for a small stimulus that is restricted to the CRF (Figures 1B–D).

Gamma-synchronization is not an all-or-nothing phenomenon, but shows a gradual dependence on the extent to which the stimulus exceeds the CRF border, with the firing statistics laying somewhere in between the highly irregular and highly gamma-rhythmic firing mode. This relationship between size and gamma-synchronicity roughly takes on a log-linear form (Figure 1D), indicating that there are diminishing returns on adding more surround data after initial information has already been added. We can explain this by the initial evidence accumulation having the greatest impact on prediction accuracy.

Besides regular textures and bar stimuli, it has been shown that other geometric patterns like colored squares, complex contours and curved lines induce strong V1 gamma-synchronization (Rols et al., 2001; Grothe et al., 2012; van Kerkoerle et al., 2014). These patterns also allow for accurate predictions of the CRF stimulus from the surround.

**Figure 2:**
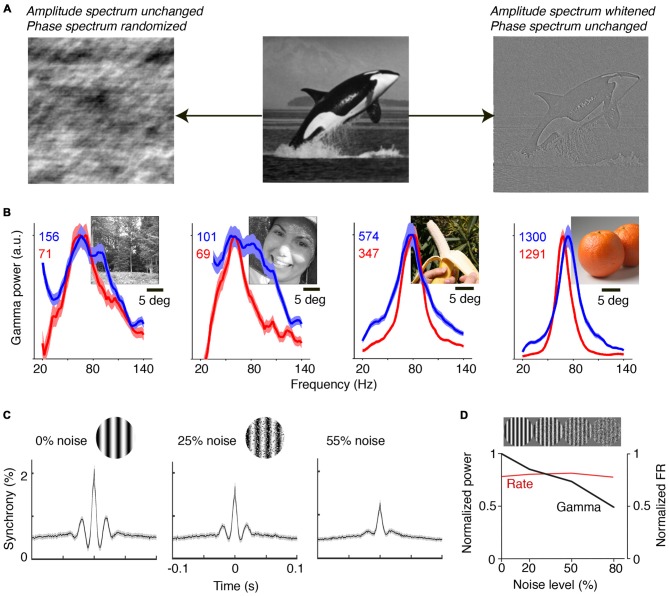
**Gamma-synchronization for natural images and noise stimuli. (A)** An image can be decomposed into the amplitude and the phase spectrum. Shown on the left is an image in which amplitude information is preserved, but where the phase spectrum is randomized. Shown on the right is an image in which phase information is preserved, but where the amplitude spectrum is whitened. The phase spectrum contains most of the meaningful image information, while the amplitude spectrum is insufficient to perform object recognition. The phase spectrum determines higher-order image correlations, like the kurtosis (4th order correlation). **(B)** V1 gamma-synchronization is reliably induced when monkeys are freely viewing natural images. LFP signals were recorded using a subdural ECoG grid and referenced bipolarly. Red and blue lines show change in LFP power spectra (as % increase) for the two monkeys separately. Narrow-band V1 gamma-synchronization was found for all 64 presented images. Adapted from Brunet et al. (2013). **(C)** Recordings from superficial layers of V1 in anesthetized cats. Visual stimuli were drifting gratings in a circular aperture of 8° diameter. Spatial noise was added to gratings by swapping randomly chosen pairs of pixel areas. Shown are average cross-correlograms across all synchronized cell pairs. Gamma-synchronization decreases with noise magnitude. Adapted from Zhou et al., 2008. **(D)** V1 recordings from superficial layers in anesthetized monkeys. Gamma LFP power (black) shows incremental decrease as a function of noise amplitude, while firing rates (FR; red) do not show a change. Adapted from Jia et al. (2013b).

Natural images contain information in both their phase and amplitude spectra. The critical component for the generation of predictions is the phase spectrum of the image, which determines most of its information content (Figure 2A). The phase spectrum defines the higher-order correlations in the image, as opposed to the amplitude spectrum, which governs the first and second-order correlations. The high-pass filtering properties in the retina effectively remove the typical 1/f^2^ structure of natural images, which has been considered as a prime example of efficient coding (Simoncelli and Olshausen, 2001; Doi et al., 2012). Thus, area V1 receives a whitened input that preserves the information in the phase spectrum. Due to the information carried in the phase spectrum, and the existence of statistical regularities in the natural visual input, natural images allow for accurate predictions of CRF content from the surround, and their viewing should thus yield substantial gamma-synchronization. Brunet et al. (2013) performed ECoG recordings from visual cortex in awake monkeys and presented a large set of natural scenes. They found narrow-band gamma-synchronization for all presented natural images (Figure 2B). The consistent observation of gamma was likely the result of the free-viewing condition: if the animal is freely viewing, it will predominantly view the locations that contain higher-order correlations, because these are known to guide fixations and increase salience (Einhäuser et al., 2006, 2008). Moreoever, it ensures that the animal samples both locations that induce strong and locations that induce weak gamma, such that gamma can, on average, be detected for each natural image. Interestingly, it appears that those natural images containing a highly homogenous, large object induced particularly strong gamma-synchronization, while those images with finer detail induced less gamma-synchronization (Figure 2B). This can be explained by the former allowing for more accurate predictions of CRF content from the surround than the latter. Recent work indicates that homogeneous natural image patches are less sparsely encoded than heterogeneous natural image patches (Coen-Cagli et al., 2015).

The ease with which gamma-synchronization can be induced using regular textures and natural stimuli stands in sharp contrast to the observed firing patterns for stimuli for which CRF content cannot be accurately predicted by the surround. Pink or Brownian noise patterns have similar power spectra as natural scenes, but lack the phase information that endow meaningful image information and that allows for accurate contextual predictions (Figure 2A). These noise patterns do not induce detectable V1 gamma-synchronization (Zhou et al., 2008; Jia et al., 2013b; Brunet et al., 2014b; Hermes et al., 2014; Figures 2C,D). In agreement with *Principle 1*, the gradual transition from a regular to a stochastic texture leads to a gradual loss of gamma. Firing rates, on the other hand, are unaffected by this transition (Jia et al., 2013b; Figure 2D).

A subtler manipulation of CRF content can also create a discrepancy between the surround and the CRF stimulus. This should, according to *Principle 1*, lead to a reduction in gamma-synchronization. In addition to gratings, Gieselmann and Thiele (2008) also presented a set of images in which there was a blank, circular annulus mask (which had the same luminance as the background) in the center of an 8° grating stimulus. A small change in CRF content with a 0.25° annulus mask did not change V1 firing rates as compared to the case of a grating. However, it did strongly reduce V1 gamma-synchronization, towards the level of a 1° grating stimulus (Figure 1B). Annulus grating stimuli with a surround but no CRF stimulus also did not induce V1 gamma-synchronization. Thus, neither CRF input alone, nor surround input alone can induce strong gamma-synchronization.

## Gamma-Synchronization and Motion Properties

The principle that accurate prediction of the CRF from the surround data results in gamma-synchronization can also be extended to stimuli with a motion component. When predictions are derived from the surround, we expect them to be based on a temporal integration of past values. This assertion makes sense not only because of the spatiotemporal statistics in the natural input, but also because synaptic inputs have to be integrated over time.

**Figure 3:**
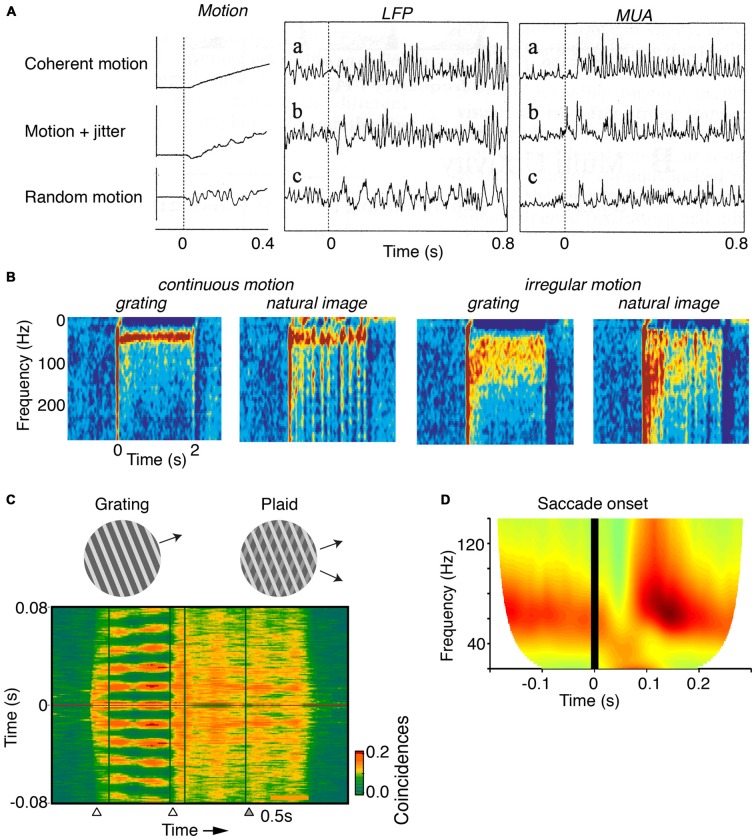
**Dependence of gamma on the motion of stimuli. (A)** V1 MUA and LFP recordings in anesthetized cats. Gamma-rhythmicity is reliably induced by a drifting grating with continuous motion. Adding random jitter to motion gradually disrupts gamma-synchronization. Adapted from Kruse and Eckhorn (1996). **(B)** V1 recordings from awake cats. Gamma-synchrony is induced when natural images or gratings have regular motion, but not when they have irregular motion. The irregular motion in this case was derived from a natural movie made from a camera mounted on a cat’s head. Adapted from Kayser et al. (2003). **(C)** V1 recordings from superficial layers in awake monkeys. Presentation of a large drifting grating (>8°) induces reliable gamma-synchronization between spiking responses recorded from different electrodes. The addition of a second grating component to a drifting grating (i.e., a plaid stimulus) reduces V1 gamma-synchronization as compared to the case of a single-component grating. Adapted from Lima et al. (2010). **(D)** V1 ECoG recordings from awake monkeys. Monkeys were freely viewing natural images. Shown is the average V1 LFP power spectrum around the saccade, as a function of time (s). Time *t* = 0 was defined as the moment of peak saccade velocity. Saccades temporarily disrupt gamma-synchronization. Adapted from Brunet et al. (2013).

Moving stimuli for which CRF data can be reliably predicted from the surround induce sustained V1 gamma-synchronization. This holds true for grating and bars with continuous and regular motion (Figures 3A,B; Gray et al., 1989; Kruse and Eckhorn, 1996; Friedman-Hill et al., 2000), but also for natural movies made from a static camera frame (Gray and Goodell, 2011; Besserve et al., 2015) or natural images on which regular motion is superimposed (Kayser et al., 2003).

In contrast, V1 gamma-synchronization is gradually reduced by superimposing an increasing amount of motion jitter onto drifting gratings (Kruse and Eckhorn, 1996; Kayser et al., 2003; Figures 3A,B). Likewise, viewing natural movies that are made from a camera mounted on the head of a moving animal, induces only weak gamma-synchronization (Kayser et al., 2003), as opposed to viewing movies made from a static camera frame (Figure 3B; Gray and Goodell, 2011; Besserve et al., 2015). This effect cannot be ascribed to the static geometric properties of the natural movie *per se*, because superimposing regular motion on individual frames from these movies does induce strong gamma-synchronization (Figure 3B; Kayser et al., 2003). Rather, the effect is due to the irregular motion that these type of natural movies have, which is demonstrated by the finding that superimposing the irregular motion flow from these movies onto a grating stimulus disrupts gamma-synchronization (Figure 3B; Kayser et al., 2003).

The extent to which CRF data can be accurately predicted by the surround can be reduced by overlaying multiple objects having incoherent motion (Gray et al., 1990; Lima et al., 2010). For example, plaid patterns diminish V1 gamma-synchronization compared to a single grating (Figure 3C), even though firing rates are not affected by this manipulation. Consistent with *Principle 1*, a gradual increase in the luminance-contrast or orientation difference between plaid components leads to a gradual decrease in gamma-synchronization (Lima et al., 2010). This result on plaid stimuli cannot be explained by the static, geometric properties of the plaid stimulus, because regular checkerboard stimuli do induce strong gamma-synchronization (Hermes et al., 2014).

Finally, it is to be expected that during saccades, there is a sudden loss of predictability of CRF data. Indeed, when monkeys freely view a natural image, there is a sudden suppression of V1 gamma-synchronization during and immediately following saccades (Figure 3D; Brunet et al., 2013). Primates make saccades at rates of about 2–4 Hz (Otero-Millan et al., 2008), which leaves ample time for gamma-synchronization to emerge after each saccade, given that gamma has a latency of about 80–100 ms (Figures 1B, 3D; but note that stimulus-locked gamma oscillations may emerge as early as 50 ms, Fries et al., 2001).

Together, these findings on the relationship between motion properties and V1 gamma-synchronization support the notion that V1 gamma-synchronization emerges whenever there is a match between predictions from the surround and the CRF.

## Stimulus-Dependence of Intercolumnar Gamma-Synchronization

In this section, we examine the properties of intercolumnar gamma-synchronization during visual stimulation. Because this principle is closely related to the BBS proposal, we first discuss the relationship of our theory with the BBS hypothesis, and proceed by examining evidence for *Principle 2*.

### Comparison to BBS

**Figure 4:**
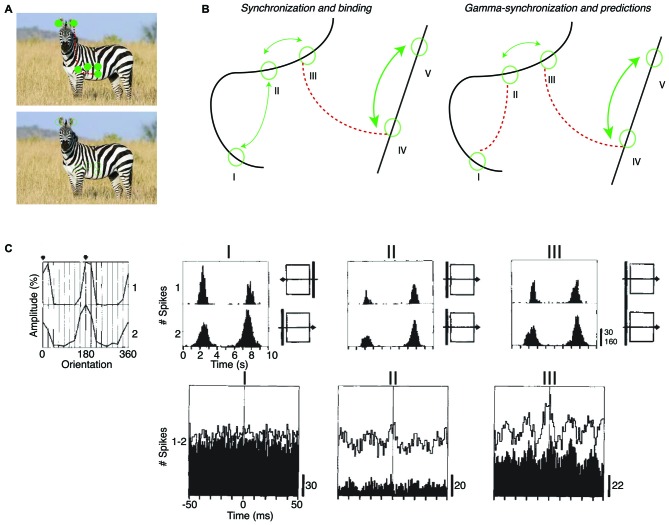
**Dependence of intercolumnar gamma-synchronization on stimulus properties. (A)** Predicted pattern of gamma-synchronization for example stimulus. Zebra has image regions with regular texture (similar to a grating) that should induce V1 gamma-synchronization when viewed from appropriate distance. Discontinuities in texture do however occur, in which case *Principle 2* predicts that multiple, incoherent gamma rhythms emerge for the zebra fur. Dashed green and dashed red connecting lines indicate the presence and absence of gamma-synchronization between neurons having non-overlapping CRFs, respectively. Top image is shown with filled circles in order to illustrate that the receptive field (RF) content can be predicted from the surround. **(B)** Schematic inspired by experiments of Gray et al. (1989) and Roelfsema et al. (2004). Green circles indicate RFs. Binding-by-synchronization (BBS) predicts gamma-synchronization between neurons responding to the same smoothly curved object (among i, ii and iii; and among iv and v), but not between neurons responding to a different object (e.g., not between iii and iv). *Principle 2* predicts gamma-synchronization only between those neurons that accurately predict each other’s visual input (i.e., between ii and iii but not i and ii). This means that there is only short-range synchronization for a stimulus like the curved line, but long-range V1 gamma-synchronization for a single, long bar stimulus. **(C)** Long-range (7 mm) V1 gamma-synchronization in anesthetized cats induced by coherently (II) moving bars, or by one single bar (III) that stimulates the CRFs of two cells simultaneously. No gamma-synchronization is detected when the bars move in opposite directions (I). Adapted from Gray et al. (1989).

*Principle 2* states that neuronal populations with non-overlapping CRFs engage in gamma-synchronization to the degree that they accurately predict each other’s visual input. This idea is closely related to the key intuition of the BBS theory, namely that V1 gamma-synchronization dynamically forms assemblies depending on structural Gestalt relationships in the visual input. It also emphasizes that the key factor governing intercolumnar gamma-synchronization is the relationship between local features in the input image. Thus, it is not surprising that many empirical findings that provide support for BBS also support *Principle 2*. Nevertheless, there are a number of important differences between *Principles 1* and *2* and BBS that we wish to clarify:

(i) *Principle 1* describes how bottom-up stimulus properties determine the precision of gamma-synchronization from the concept of predictability of CRF data from the surround. This accounts for several aspects of gamma-synchronization, namely: (i) The dependence of gamma-synchronization on the size of an object (see “The Relationship Between Gamma-Synchronization and Geometry” Section; Figure 1B), (ii) the dependence of gamma on the texture of an object (see “The Relationship Between Gamma-Synchronization and Geometry” Section; Figure 2), and (iii) the dependence of gamma on the motion of an object (see “The Relationship Between Gamma-Synchronization and Motion Properties” Section; Figure 3). We believe that, from the perspective of the BBS hypothesis, it is difficult to explain why gamma-synchronization between cells responding to the same object is not invariant to these properties.

(ii) According to *Principle 2*, mutual predictability rather than perceptual grouping or binding is the main criterion for intercolumnar gamma-synchronization. This leads to some overlapping, but also some different predictions for intercolumnar gamma-synchronization. BBS predicts, for example, that cells responding to the same smooth contour fire in synchrony (Figure 4B). *Principle 2* predicts that only a subset of neurons responding to the same object might gamma-synchronize, to the degree that they mutually predict each other’s visual input. We also predict that some objects might be accompanied by a complex pattern of gamma-synchronization, with multiple gamma-rhythms that are only weakly coherent (Figure 4A).

(iii) BBS considers gamma-synchrony as a potential solution to the binding or scene segmentation problem, while we seek the functional role of gamma-synchronization in the context of efficient coding (see “Functional Consequences of Gamma-Synchronization” Section).

### Empirical Support for Principle 2

Long-range V1 gamma-synchronization between columns has been observed conform to *Principle 2*. In their first report about gamma-synchronization, Gray et al. (1989) already observed that there is long-range zero-lag gamma-synchronization between two V1 cells with non-overlapping CRFs when they are stimulated by two separate bars having motion congruence (Gray et al., 1989; Figure 3A). This inter-columnar gamma-synchronization was detected up to 8 mm distance. Further, abundant zero-lag gamma-synchronization between sites with non-overlapping RFs has been observed when they are stimulated by the same regular texture, e.g., a grating (Gail et al., 2000; Maldonado et al., 2000; Ray and Maunsell, 2010), or by the same bar stimulus (Gray et al., 1989; Livingstone, 1996; Figure 4C). These findings suggest that when neuronal populations with non-overlapping CRFs can predict each other’s visual input, they engage in gamma-synchronization.

The predictability between visual inputs will be reduced when two neurons with non-overlapping CRFs are stimulated by two separate parallel bars as compared to the case of one long bar. This is paralleled by a reduction in gamma-synchronization for V1-V1 cell pairs and V1-PLMS (an extrastriate region) cell pairs in anesthetized cat (Gray et al., 1989; Engel et al., 1991), and V1-V1 cell pairs in anesthetized squirrel monkey (Livingstone, 1996; Figure 4C). Because of the natural statistics of visual input, we expect that predictability is further reduced when the two separate bars move in opposite directions, although the cortex may rapidly update, through stimulus repetition, the expected statistical relationships between visual inputs (Brunet et al., 2014a; see “Gamma-Synchronization Depends on Experience and Development” Section). Again, this is paralleled by either a loss (V1-V1 and V1-PLMS in anesthetized cats; Gray et al., 1989; Engel et al., 1991) or reduction (V1-V1 in anesthetized squirrel; Livingstone, 1996) in gamma-synchronization (Figure 4C). Later studies also demonstrated that when a regular texture is divided into two contours by a phase offset, there is strongly reduced V1 gamma-synchrony between cells with CRFs covering the separate contours, compared to the case of a continuous texture (Gail et al., 2000; Biederlack et al., 2006).

Only one study has explicitly compared the strength of V1 synchronization between cells responding to the same object and cells responding to different objects. Roelfsema et al. (2004) made recordings from V1 of awake monkeys performing a curve-tracing task, presenting two curved lines simultaneously. They found that neurons having CRFs covering distant part of the same smooth, curved line showed no detectable gamma-synchronization. Roelfsema et al. (2004) also found that there was no increased zero-lag synchrony when the two neurons had CRFs covering the same curved line, as compared to the case when two neurons had CRFs covering two different curved lines (Figure 4B). They did find that the cells having CRFs on the same curved line showed more covariation of firing rates than cells having their CRF on different curved lines (Roelfsema et al., 2004). This was taken as direct evidence against the BBS hypothesis, and led Roelfsema et al. (2004) to suggest that perceptual grouping and binding do not rely on gamma-synchronization. However, Roelfsema et al. (2004) did report that there was gamma-synchronization between cells that had CRFs in close proximity, which is consistent with later reports using similar stimuli (van Kerkoerle et al., 2014). For the curved line stimulus, *Principle 2* can explain the strong dependence of gamma-synchronization on distance, because those cells with nearby CRFs would receive visual inputs that predict each other accurately, whereas cells with distant CRFs would not predict each other’s visual input (Figure 4B). Although these data support our hypothesis, it needs to be noted that the data of Roelfsema et al. (2004) should be interpreted with some caution because they were biased towards deeper layers, which tend to have weaker gamma-synchronization (Gray et al., 1990; Livingstone, 1996; Buffalo et al., 2011; see “Laminar Dependence of Gamma-Synchronization” Section).

In sum, only few experiments have been performed that directly test the predictions of *Principle 2*. While the results of these studies are largely consistent with *Principle 2*, future experiments are needed to probe the exact properties of intercolumnar gamma-synchronization.

## Mechanisms of Gamma-Synchronization

The data reviewed above suggests that the mere activation of a cluster of cortical columns is necessary, but by itself insufficient, to generate strong gamma-synchronization (Figure 1B). This point calls for a revision of canonical models of gamma generation, in which the critical factor is the drive to a local network containing inhibitory and excitatory cells (Whittington et al., 1995, 2000; Bartos et al., 2007; Cardin et al., 2009; Tiesinga and Sejnowski, 2009; Buzsáki and Wang, 2012). In particular, the specific pattern of spatially distributed zero-lag gamma-synchronization between cortical columns suggests that recurrent dynamics through excitatory feedback (i.e., axonal projections from extrastriate cortex to V1) and lateral connections are likely critical for the emergence of V1 gamma-synchronization. In what follows, we will discuss the relationship between the stimulus dependencies of gamma-synchronization and its underlying mechanisms.

### Roles of Lateral and Extrastriate Feedback Connections

#### Feedback Connections

**Figure 5:**
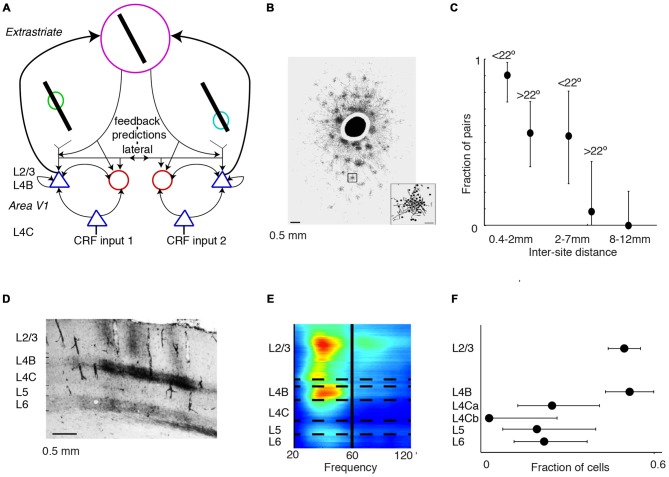
**Mechanisms of gamma-synchronization: connections and lamina. (A)** Schematic that illustrates various aspects of the V1 circuit. Cells receive information from the surround through lateral and extrastriate feedback projections that carry predictions and are predominantly excitatory. These projections target both excitatory and inhibitory cells in L4B and L2/3. The surround inputs are processed within the local column through recurrent excitatory and inhibitory interactions. L4B and L2/3 also receive bottom-up inputs from L4C, which does not receive substantial extrastriate feedback and lateral input. **(B)** Patchy pattern of axonal projections in V1 of squirrel monkey. Scale bar is 0.5 mm. Black circle corresponds to injection site. Adapted from Angelucci et al. (2002b). **(C)** Fraction of pairs showing synchrony for different distances and preferred orientation differences during presentation of a moving bar stimulus. Error bars show 95% confidence intervals derived from binomial distribution. Recordings were made from V1 of anesthetized cats. Adapted from Gray et al. (1989). **(D)** Horizontal connectivity in V1 of squirrel monkey. Scale bar is 0.5 mm. Injection site to the left (not shown). Patchy connectivity pattern is visible in L2/3 and L4B, but mostly absent in L4C and L5. Connectivity is also observed with L6. Adapted from Rockland and Lund (1983). **(E)** Laminar recordings from anesthetized macaque monkeys. Gamma LFP power is most prominent in L2/3 and L4B. Adapted from Xing et al. (2012). **(F)** Fraction of V1 cells showing gamma-rhythmic firing in > 25% of responses. The 95% confidence intervals were derived by using the binomial distribution. Recordings were made from anesthetized squirrel monkeys. Lamina were identified through *post mortem* histological analysis performed after each experiment (16 monkeys). V1 gamma-synchronization is most prominent in L2/3 and L4B, and weaker in L4C and L5/6. Adapted from Livingstone (1996).

Visual input in the surround can affect neuronal firing and gamma-synchronization in area V1 both through extrastriate feedback and lateral connections (Figure 5A). Thus, both might potentially contribute to the generation of gamma-synchronization. It appears unlikely that neurons in area V2 directly synchronize the gamma phases between V1 columns, for the following two reasons. First, gamma-synchronization between areas V1 and V2 is largely restricted to V1 and L4/deep L3 of V2, following the pattern of anatomical projections from superficial V1 layers to area V2 (Rockland and Virga, 1990; Roberts et al., 2013; Zandvakili and Kohn, 2015; see also “Gamma Across the Visual Hierarchy” Section). However, extrastriate feedback originates from infragranular and superficial, but not granular layers (Rockland and Virga, 1989; Anderson and Martin, 2009). Thus, it appears that area V2 provides an input to area V1 that is not strongly gamma-synchronized with V1 activity, which can by itself not cause a phase-coherent entrainment of different V1 sites. Second, Granger-causality analyses demonstrate that gamma-coherence has a characteristic feedforward V1-to-extrastriate cortex (V2, V4) signature (Bosman et al., 2012; van Kerkoerle et al., 2014; Bastos et al., 2015a), and activity in area V1 leads activity in area V2 by a few milliseconds (Jia et al., 2013a; Zandvakili and Kohn, 2015). Yet, a feedback signature would be expected if extrastriate cortex would be the main source driving V1 gamma-synchronization. Nevertheless, even if signals from extrastriate cortex do not directly gamma-synchronize different V1 sites, they might nonetheless modulate the strength of V1 gamma-synchronization. This would especially hold true for modulations of gamma-synchronization caused by visual stimulation in the far surround, because cortical feedback is required for suppression of neuronal firing rates by the far surround (Angelucci et al., 2002a,b).

#### Lateral Connections

These considerations on the role of extrastriate feedback lead to the prediction that the emergence of V1 gamma-synchronization depends strongly on the lateral, recurrent connectivity within area V1. In area V1, there exists an extensive lattice of recurrent, patchy connections (Figure 5B; Gilbert and Wiesel, 1983; Rockland and Lund, 1983; Lund et al., 2003) with axons that are thick (~1–3 micron) and myelinated, presumably having high conduction velocities corresponding to axonal delays on the order of a few milliseconds (Kisvarday and Eysel, 1992). This network extends, both in cats and monkeys, to about 7 mm on average (up to ~10 mm) and carries information over several degrees of visual field, covering an area that is several times the CRF size (Gilbert and Wiesel, 1983; Rockland and Lund, 1983; Kisvarday and Eysel, 1992; Angelucci et al., 2002a,b; Lund et al., 2003; Figure 5B). These long-range excitatory projections target both excitatory and inhibitory cells, and project most strongly to cells with the same orientation preference (Gilbert and Wiesel, 1989; Lund et al., 2003). Thus, this patchy network with its widespread recurrent connections is a plausible substrate to maintain V1 gamma-synchronization. Consistent with this idea, Gray et al. (1989) found that long-range (intra-hemispheric) V1 gamma-synchronization was restricted to a scale of <7–8 mm (Figure 5C), i.e., to pairs of neurons that could have received a common projection within the same patchy network lattice (Angelucci et al., 2002a,b). Gray et al. (1989) further showed that on the 2–7 mm scale, gamma-synchronization was restricted to pairs of neurons with overlapping orientation preferences (Gray et al., 1989; Figure 5C). In addition, the prevalence of gamma-synchronization between neurons with a similar orientation preference might result from their ability to predict each other’s input. Finally, in agreement with the spatial spread of the patchy connections (Angelucci et al., 2002a,b), manipulations of stimulus size show that gamma-synchronization in awake monkeys tends to saturate around 3° (Gieselmann and Thiele, 2008; Ray et al., 2013). Thus, we conclude that V1 gamma-synchronization likely depends on recurrent, long-range interactions over the patchy axonal network.

### Laminar Dependence of Gamma-Synchronization

The importance of lateral connections for the generation of gamma-synchronization might shed light on the laminar distribution of gamma-synchronization. Anatomical evidence suggests that within the parvocellular pathway, layer 2/3 circuitry is well endowed to generate gamma-synchronization based on the integration between surround and bottom-up inputs. L2/3 inhibitory and excitatory cells receive a mixture of both bottom-up L4C inputs (the CRF input) and surround data through an extensive web of horizontal connections or feedback from extrastriate cortex (Gilbert and Wiesel, 1983; Rockland and Lund, 1983; Burkhalter and Bernardo, 1989; Angelucci et al., 2002a,b; Lund et al., 2003; Figure 5D). On the other hand, input layer L4C lacks an extensive network of horizontal projections, and extrastriate feedback also skips this layer (Rockland and Lund, 1983; Burkhalter and Bernardo, 1989; Angelucci et al., 2002a,b; Lund et al., 2003). Horizontal connections and feedback are less prominent in layer 5, which also receives no direct input from L4C (Angelucci et al., 2002a,b; Lund et al., 2003). Thus, L2/3 receives the necessary information to compare the CRF input with the predictions from the surround. Consistent with these functional cytoarchitectonic features, gamma-synchronization has been demonstrated to be stronger in superficial layers than in L4C and infragranular layers (Gray et al., 1990; Livingstone, 1996; Buffalo et al., 2011; Hansen and Dragoi, 2011; Figures 5E,F).

L4B is the end-point of the exclusive magnocellular pathway in V1 and projects directly to extrastriate cortex, but also receives extrastriate feedback (Rockland and Lund, 1983; Burkhalter and Bernardo, 1989; Lund et al., 2003). Similar to L2/3, L4B also integrates L4C input with surround inputs that it receives through extensive horizontal connections (Rockland and Lund, 1983; Burkhalter and Bernardo, 1989; Lund et al., 2003). There is evidence that, for moving stimuli, L4B activity is strongly gamma-rhythmic (Livingstone, 1996). Likewise, laminar recordings reveal that LFP power exhibits two spatially separated gamma peaks in L2/3 and L4B (Xing et al., 2012; Figures 5E,F). Thus, gamma-synchronization in L4B might be generated according to the same principles as in L2/3, except that it would depend more strongly on motion properties than L2/3 gamma.

The prevalence of gamma-synchronization in those layers projecting heavily to extrastriate regions (L4B and L2/3) is consistent with a series of recent findings showing that the gamma-frequency band is associated with the feedforward transmission of information (Bosman et al., 2012; van Kerkoerle et al., 2014; Bastos et al., 2015a; Michalareas et al., 2016).

### GABAergic Inhibition and Surround Suppression

In this section, we will review the role of GABAergic inhibition and surround suppression in the generation of gamma-synchronization.

**Figure 6:**
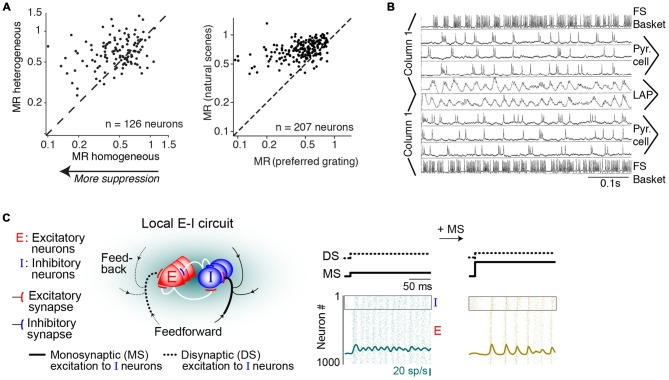
**Mechanisms of gamma-synchronization: surround suppression and network dynamics. (A)** Different stimuli produce various degrees of surround suppression in area V1. Homogenous image patches in natural scenes are associated with more surround suppression than heterogeneous image patches. Drifting gratings are associated with more surround suppression than natural scenes. Adapted from Coen-Cagli et al. (2015). **(B)** Network model of gamma-synchronization between two columns that have weak excitatory projections to one another, targeting both pyramidal cells and fast spiking (FS) basket cells. For conduction delays <5 ms, the network produces intercolumnar gamma-synchronization. Adapted from Bush and Sejnowski (1996). **(C)** Network model of gamma-synchronization and its modulation by the MS-DS ratio. Inhibitory interneurons receive both monosynaptic and disynaptic (DS; i.e., mediated by local excitatory neurons) inputs. The MS-DS ratio governs the strength of gamma-synchronization. Adapted from Jadi and Sejnowski (2014).

Surround stimulation leads to a sparsification of the firing rates of excitatory cells (Figures 1B, 6A). This sparsification may reflect efficient coding of visual input, because the entropy inequality H(S_CRF_|X_Surround_) ≤ H(S_CRF_) indicates that we need less bits to encode a stimulus when we have knowledge from another variable (in this case surround input data). Sparsification also plays a role in a predictive coding scheme, because only the prediction error needs to be transmitted between nodes, rather than the stimulus estimate itself (Rao and Ballard, 1999; Bastos et al., 2012).

Both surround suppression and gamma-synchronization are strongly associated with GABAergic inhibition. While the firing rate of excitatory neurons decreases strongly with surround stimulation, the firing rate of GABAergic interneurons increases or only slightly decreases when a natural stimulus extends beyond the CRF border (Haider et al., 2010; Adesnik et al., 2012; Pecka et al., 2014). It is commonly thought that GABAergic interneurons, through their interactions with excitatory cells, play a critical role in the generation of gamma-synchronization in both hippocampus and neocortex (Whittington et al., 1995, 2000, 2011; Hasenstaub et al., 2005; Bartos et al., 2007; Cardin et al., 2009; Tiesinga and Sejnowski, 2009; Buzsáki and Wang, 2012; Moca et al., 2014; Salkoff et al., 2015). Yet, the precise contribution of GABAergic interneurons to gamma-synchronization in visual cortex remains largely unclear. Data from area V4 in awake monkey (Vinck et al., 2013a) and rodent V1 (Vinck et al., 2015, 2016; Perrenoud et al., 2016) have revealed stronger gamma-synchronization in putative/identified FS, GABAergic interneurons than putative/identified excitatory cells. Yet, gamma-synchronization in these areas and species may have different properties than in area V1 of carnivores and primates (see “Gamma Across the Visual Hierarchy” Section). Moreover, a study performing intracellular recordings from FS, GABAergic interneurons in area V1 of anesthetized cats did not find gamma-rhythmicity in their spiking responses (Azouz et al., 1997).

Based on the association of both surround suppression and gamma-synchronization with GABAergic inhibition, it has been conjectured that gamma-synchronization results from the recruitment of GABAergic interneurons through surround inputs (Gieselmann and Thiele, 2008; Jadi and Sejnowski, 2014). We will argue however that surround suppression consists of two separate components, out of which only one component is strongly associated with gamma-synchronization:

A first “untuned” suppression component has a much earlier onset (~60 ms) than gamma-synchronization, which has an onset latency of about 80–100 ms (Figure 1B). This first suppression component normalizes unselectively for gross population activity and is independent of the relationships between CRF and surround (Xing et al., 2005; Smith et al., 2006). This type of surround suppression can be observed for cross-oriented surrounds or heterogeneous natural image patches (Xing et al., 2005; Coen-Cagli et al., 2015). It also occurs for stimuli that do not induce gamma-synchronization at all, like white noise (Vinje and Gallant, 2000; Freeman et al., 2013). A possible function of this type of surround suppression is to hold the overall excitatory input drive that a neuron in a downstream visual area receives from V1 relatively constant and invariant of stimulus size. This size-invariance could also prevent large objects from biasing the competition among multiple objects that are encompassed by the larger RFs of neurons in extrastriate cortex (Desimone and Duncan, 1995; Reynolds et al., 1999).

A second “predictive” component would reflect efficient coding by removing statistical redundancies between the CRF and surround. This second type of surround suppression can be observed for iso-oriented surrounds or homogenous natural image patches (Xing et al., 2005; Coen-Cagli et al., 2015; Figure 6A), i.e., stimuli that also induce strong gamma-synchronization. Thus, gamma-synchronization should always be accompanied by this type of predictive surround suppression. This surround suppression component has a late onset (Xing et al., 2005; Coen-Cagli et al., 2015; ~80 ms), just like gamma-synchronization (Figure 1B). The later onset of the second type of surround suppression and the gamma that accompanies it might relate to the computational complexity of the CRF-surround integration, in which multiple likelihood equations need to be solved through recurrent prediction and updating (Rao and Ballard, 1999).

### Excitatory Cell Responses

The hypothesis that the patchy, long-range axonal projections play an important role in the generation of gamma-synchronization points to the importance of excitatory cells for sustaining gamma-synchronization. There is substantial diversity in excitatory cell type responses both *in vitro* and *in vivo*. These include chattering cell responses, irregular bursting and regular spiking (Gray and McCormick, 1996; Steriade et al., 2001; Nowak et al., 2003). The responses of excitatory cells that are phase locked to gamma have been described as “bursty” both in anesthetized and awake monkeys (Gray et al., 1990; Livingstone, 1996; Gray and Viana Di Prisco, 1997; Friedman-Hill et al., 2000). It should be noted that a regular spiking cell whose output is described by a non-homogenous, gamma-rhythmic Poisson process will tend to produce a spiking output that appears bursty, at least if the neuron’s refractory period is short. However, there exists a subclass of pyramidal cells (“chattering cells”) that does have intrinsic resonance in the gamma-frequency band. When activated by direct current injections or visual stimulation, these cells tend to produce bursts of spikes at the intervals of a gamma cycle (Gray and McCormick, 1996). These chattering cells thus generate a highly gamma-rhythmic output, and there is evidence that they also receive strongly gamma-rhythmic inputs (Gray and McCormick, 1996; Cardin et al., 2005). Chattering cells are mostly found in L2/3 and have axonal projections to other columns (Gray and McCormick, 1996), enabling them to provide an excitatory, gamma-rhythmic driving source to other columns. Thus, pyramidal cells with chattering cell response properties might be specialized in encoding and transmitting information in cases where the surround accurately predicts the CRF input.

### Network Dynamics

We have reviewed which circuit elements are involved in the generation of gamma-synchronization, and now ask through which network dynamics these circuit elements generate gamma-synchronization. Bush and Sejnowski (1996) have shown that gamma-synchronization can be maintained between two separate neuronal populations that are connected through weak excitatory projections. They simulated a network comprising two columns with sparse, bidirectional excitatory projections, targeting both excitatory and inhibitory (FS basket) cells (Bush and Sejnowski, 1996; Figure 6B). This network generated robust zero-lag gamma-synchronization when conduction delays were <5 ms (Figure 6B; Bush and Sejnowski, 1996). Critical in this model was the feedforward projection to inhibitory FS basket cells inducing negative feedback (Bush and Sejnowski, 1996), which is consistent with their purported role in gamma-rhythmogenesis, as reviewed in “GABAergic Inhibition and Surround Suppression” Section.

Jadi and Sejnowski (2014) further examined the mechanisms underlying gamma-synchronization in a network containing local excitatory (E_local_) and inhibitory (I) neurons, with recurrent interactions between and within the E_local_ and I populations (Figure 6C). In this model, both E_local_ and I neurons received a tonic, monosynaptic excitatory drive from external input. Thus, inhibitory neurons received excitation that was either monosynaptic (MS; E_external_>I) or disynaptic (DS), i.e., mediated by local excitatory neurons (E_external_>E_local_>I). Jadi and Sejnowski (2014) found that the strength of gamma-synchronization was commensurate with the MS over DS ratio (i.e., with relatively stronger MS inputs; Figure 6C). The MS-DS ratio might be an important factor explaining the variation in gamma-synchronization with surround stimulation *in vivo*. Presumably, surround inputs cause a shift in the MS over DS ratio because: (i) they cause a strong decrease in the activity of local L2/3 pyramidal cells (see “GABAergic Inhibition and Surround Suppression” Section), which reduces the DS inputs, and (ii) L2/3 GABAergic interneurons likely receive increased MS drive from the surround (Haider et al., 2010; Adesnik et al., 2012; Pecka et al., 2014).

Several studies have suggested that perisomatically targeting, FS GABAergic interneurons are critical for the generation of gamma-synchronization (Bartos et al., 2007; Cardin et al., 2009; Sohal et al., 2009; Perrenoud et al., 2016; but see Azouz et al., 1997). Yet, these cells receive a strong monosynaptic input from L4 (Helmstaedter et al., 2008; Adesnik et al., 2012), which, considering the model of Jadi and Sejnowski (2014), raises the question why gamma-synchronization is not reliably induced by CRF stimulation alone. A possibility is that besides the perisomatically targeting PV (Parvalbumin positive) cells, other GABAergic interneurons are important for gamma-rhythmogenesis. Adesnik et al. (2012) have shown that in awake mice, L2/3 dendrite-targeting SOM (Somatostatin positive) cells strongly increase their firing rate with visual stimulation in the surround and contribute to the surround suppression of L2/3 pyramidal cells. In rodents, these SOM cells do not receive a substantial feedforward projection from L4 and are strongly activated by lateral excitation (Helmstaedter et al., 2008; Adesnik et al., 2012). Thus, the MS-DS ratio of these cells should strongly increase with surround stimulation. Interestingly, in L2/3 of rodent barrel cortex, many of these SOM cells exhibit FS properties, similar to PV interneurons (Gentet et al., 2012; Vinck et al., 2016). These findings suggest a potential role for L2/3 SOM cells in gamma-rhythmogenesis, although it remains unknown whether they extend to carnivores and primates.

Another open question is why surround suppression by itself is not sufficient for the generation of gamma-synchronization (see “The Relationship Between Gamma-Synchronization and Geometry” and “GABAergic Inhibition and Surround Suppression” Section). One possible explanation for the lack of gamma-synchronization with some forms of surround stimulation is that the firing of GABAergic interneurons and gamma-resonant excitatory cells with chattering response properties (Gray and McCormick, 1996; Haider et al., 2010), is particularly dependent on the relationship between CRF and surround input. This would predict that there exists a specific pattern of synaptic weights from lateral L2/3 inputs and bottom-up L4 inputs onto L2/3 excitatory and inhibitory neurons, which allows these cells to detect a match between surround and bottom-up CRF input. The finding that FS interneuron firing rates increase when CRF and surround are costimulated by natural movies suggests that this might indeed be the case (Haider et al., 2010). Likewise, we expect that, dependent on the relationship between CRF and surround stimulation, excitatory surround inputs actively contribute to the generation of spiking in L2/3 excitatory cells, even though their firing rates decrease with surround stimulation. We expect this to hold true for two reasons. First, because the integration of predictions from the surround with CRF data should lead to an adjustment of stimulus likelihood representations as expressed by the firing rates of these cells (Rao and Ballard, 1999; see “Relationship Between Gamma-Synchronization and Firing Rate Coding” Section). Second, because the emergence of intercolumnar gamma-synchronization likely depends on long-range E-E projections that temporarily increase the probability of spiking in excitatory cells before strong feedback inhibition kicks in Bush and Sejnowski (1996).

## Gamma-Synchronization Depends on Experience and Development

The mechanisms and stimulus correlates of gamma reviewed above highlight the importance of lateral connections. These lateral connections are a plausible candidate for storing the natural image statistics. Knowledge of the natural image statistics might partially depend on experience. Based on *Principle 1*, we hypothesize that gamma-synchronization is an experience-dependent phenomenon whose strength in general increases as learning increases the accuracy of surround predictions.

**Figure 7:**
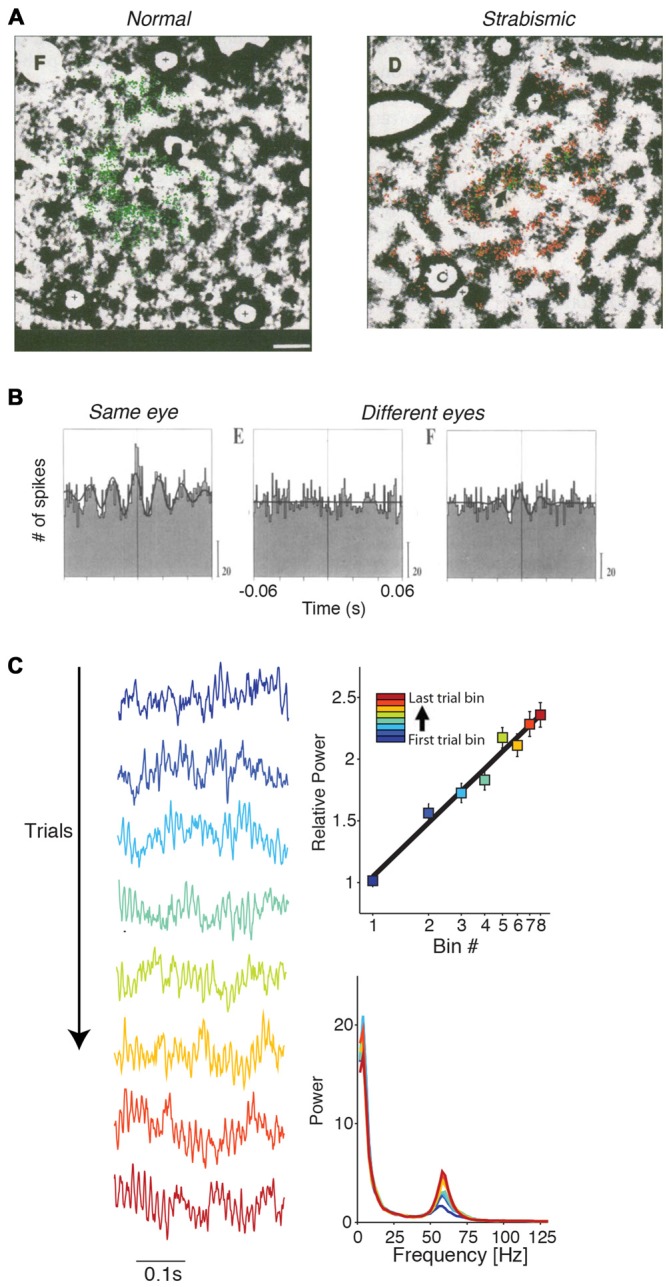
**Dependence of gamma-synchronization on experience and development. (A)** In strabismic cats (right), tangential connections between cells responding to different eyes are strongly reduced in comparison to normal case (left). Shown is a tangential section. Green and red dots correspond to retrogradely labeled cells using either green or red beads injections. Scale bar is 1 mm. Adapted from Löwel and Singer (1992). **(B)** V1 gamma-synchronization between cells responding to inputs from different eyes is strongly reduced in strabismic cats in comparison to normal case. Adapted from König et al. (1993). **(C)** Gamma-synchronization increases with stimulus presentation, both in areas V1 and V4 of the awake monkey. Recordings were made with subdural ECoG grid. Trials were divided into eight equispaced bins. On the left, LFP traces are shown for each of the eight bins for an example recording site. On the top right shown the % change in gamma power for this example site (52–74 Hz). Right bottom shows the raw power spectrum (a.u.) for the eight bins for this example site. Adapted from Brunet et al. (2014a).

Lateral cortical connections develop after birth and their selectivity is experience-dependent and achieved by pruning (Luhmann et al., 1986, 1990; Callaway and Katz, 1990, 1991; Ko et al., 2013; Cossell et al., 2015). Löwel and Singer (1992) investigated the experience-dependence of these lateral connections by rendering cats strabismic at 2–3 weeks age, which decorrelates the inputs from two eyes. Subsequently, lateral connections became specific to cells activated by input from the same eye, i.e., specific to those neuronal populations receiving correlated inputs (Figure 7A; Löwel and Singer, 1992). Using the same paradigm, König et al. (1993) showed that gamma-synchronization also became specific to pairs of cells that were activated by the same eye (Figure 7B; König et al., 1993). These finding suggests that the emergence of V1 gamma-synchronization is dependent on learning the natural statistics of visual input through experience, and that this depends on a modification of lateral connections.

In addition, there exists evidence that gamma-synchronization can be modified by experience on a faster time-scale. Both V1 and V4 gamma-synchronization tend to increase log-linearly with trial presentation number (Brunet et al., 2014a; Figure 7C). This finding suggests that over time, the match between surround predictions and CRF-inputs increases, presumably because the network updates the expected statistical relationship between surround and CRF input.

## Gamma Across the Visual Hierarchy

### Gamma-Synchronization in Higher Visual Areas

**Figure 8:**
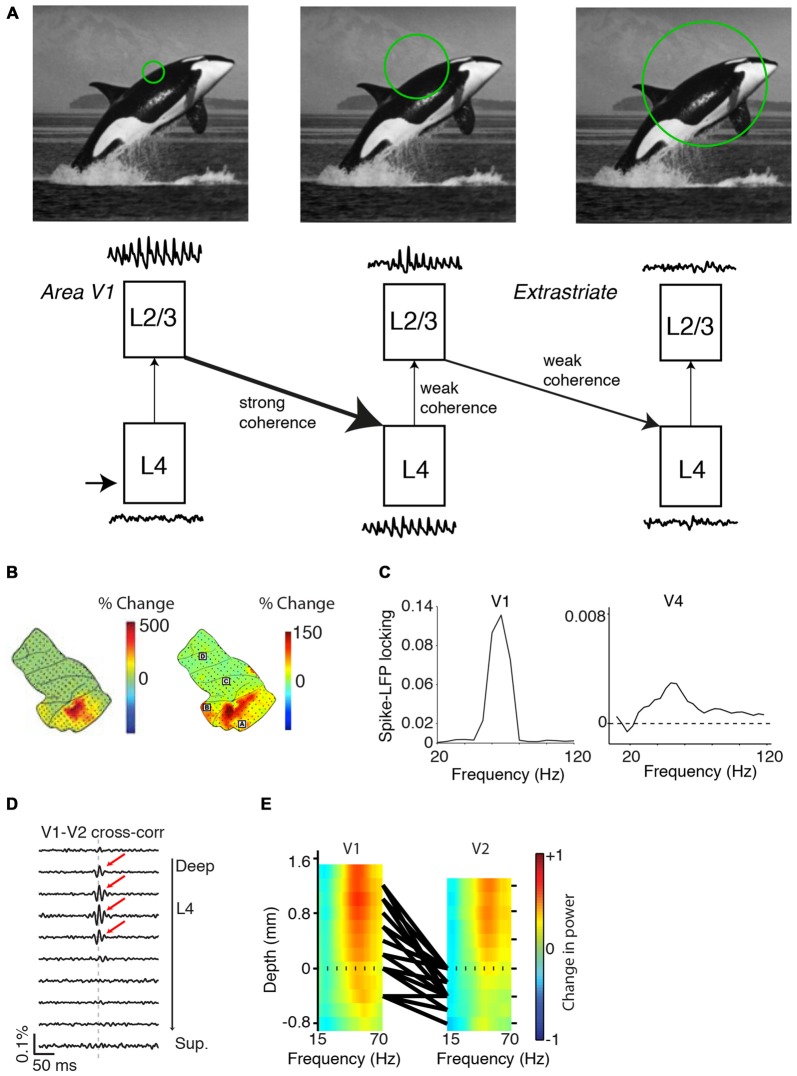
**Variability in gamma-synchronization across areas and species. (A)** Schematic overview of how the strength of gamma-synchronization differs across visual areas, and of the extent to which gamma propagates. L4C in area V1 provides an irregular spiking input to L2/3 in V1, which transforms this input into a gamma-rhythmic output provided that CRF content is accurately predicted by the surround. This gamma-rhythmic output can then entrain L4 in downstream visual areas, in which neurons have larger RFs. L2/3 in the next visual area can generates a gamma-rhythm that is only weakly synchronous with the gamma-rhythm in L2/3 of area V1 (see “Gamma Across the Visual Hierarchy” Section). Because of the larger RF size (see “Gamma Across the Visual Hierarchy” Section), gamma-synchronization is on average weaker in L2/3 of higher than lower visual areas. This will also cause a weaker entrainment of L4 in the next downstream visual area. **(B)** Spatial topography of increases in gamma LFP power for grating stimuli (left) and natural stimuli (right). Increases in LFP gamma power are stronger in area V1 and weaker in downstream visual areas. Adapted from Brunet et al. (2014a, 2013). **(C)** Left: spike-field locking (estimated with a metric not biased by firing rate or spike count; Vinck et al., 2012) in area V1 of the awake monkey. Adapted from Womelsdorf et al. (2012). Right: spike-field locking of L2/3 pyramidal cells in area V4 of the awake monkey. Adapted from Vinck et al. (2013a). **(D)** V1-V2 cross-correlations, recorded from anesthetized monkeys during presentation of drifting grating stimuli. Cross-correlograms with zero-lag peaks were mostly restricted to V1 and middle-layer V2 pairs. Adapted from Zandvakili and Kohn (2015). **(E)** Laminar recordings from areas V1 and V2. Colormaps show the induced LFP power as a function of frequency. Induced LFP power was defined as (S−B)/(S+B), where S and B are the LFP power during visual stimulation and baseline, respectively. Monkeys viewed static grating stimuli that had an average diameter of 5° and 72% luminance-contrast. Black lines correspond to the top 5% pairs with strongest CSD-CSD (current source density) gamma-coherence. Dashed line indicates top layer 4C in area V1 and top layer 4 in area V2. Strongest gamma-band coherence is seen between the output layer (L2/3) of V1 and the input layer (L4) of V2. Adapted from Roberts et al. (2013).

It is possible that the principles underlying the stimulus dependencies of gamma-synchronization in area V1 also apply to higher visual areas that are retinotopically organized. Compared to area V1, cells in higher visual areas exhibit more complex and non-linear response properties, as well as larger RFs. If gamma-synchronization in higher visual areas would also be generated according to *Principles 1* and *2*, then it is required that accurate predictions of CRF from surround data can be made on larger spatial scales. Thus, we expect that the gamma vs. size dependency curve (Figure 1D) shifts rightwards. In addition, it is to be expected that the statistical correlations in natural visual input fall off as a function of retinotopic distance. Hence, although powerful gamma-synchronization has been demonstrated to exist in many extrastriate regions of the cat and the primate visual cortex (Engel et al., 1991; Fries et al., 2001; Buffalo et al., 2011), we predict that gamma-synchronization in L2/3 of higher visual areas is typically weaker than in area V1. Thus we expect the gamma vs. size dependency curve to shift rightwards, and to scale downwards. In general, we hypothesize that gamma-synchronization shows a negative dependence on RF size. There is empirical evidence for this hypothesis (i) across areas, (ii) within V1 and (iii) across species:

(i) Data from ECoG and laminar recordings during visual stimulation with gratings or natural images reveal that gamma-power increments are stronger in area V1 as compared to higher visual areas (which were simultaneously recorded; Figures 8B,E). Further, reported spike-field phase locking values of isolated single units are about 10–30× higher in e.g., area V1 than area V4 (Figure 8C; Womelsdorf et al., 2012; Vinck et al., 2013a). An examination of studies using metrics of spike-field coherence (SFC) based on multi-unit data yield more mixed results. Chalk et al. (2010) made simultaneous recordings from areas V1 and V4 in awake monkeys and reported SFC values between multi-unit and LFP that were about twice as high in area V1 than in area V4. On the other hand, Buffalo et al. (2011) reported SFC values between V1, V2 and V4. While V1 SFC was higher than in V4, V2 SFC tended to be higher than in V1. We discuss problems with the interpretation of this comparison in the “Appendix” Section.

(ii) Gamma-synchronization tends to be stronger around foveal than large eccentricities in area V1, presumably because CRF size increases towards the periphery (van Pelt and Fries, 2013).

(iii) It can be predicted that in species having large V1 receptive fields RFs (such as mouse), gamma is considerably weaker than in area V1 of carnivores and primates. During visual stimulation, gamma phase locking values (when measured with unbiased metrics) and LFP power changes (as compared to baseline) are indeed considerably smaller in mouse than in primate and cat V1 (Niell and Stryker, 2008; Vinck et al., 2015, 2016; Perrenoud et al., 2016).

### Propagation of Gamma Throughout the Visual System

In this section, we will argue that there exist a granular-layer and a superficial-layer gamma that are generated through different mechanisms, and explore the interaction between these two types of gamma.

Gamma-synchronization in L2/3 of the upstream area can entrain L4 cells in the downstream area (Figures 8D,E; Roberts et al., 2013; Zandvakili and Kohn, 2015). There is evidence that in the downstream area, this L4 feedforward gamma may not propagate effectively to the superficial layers (Figure 8D; Zandvakili and Kohn, 2015), although this likely depends on stimulus properties and attentional/behavioral state (see below). Zandvakili and Kohn (2015) found prominent V1-V2 zero-lag cross-correlation peaks between superficial V1 and middle V2 layers, but not between superficial V1 and superficial V2 layers. Roberts et al. (2013) performed simultaneous, laminar LFP recordings from areas V1 and V2 (Figure 8E). While for both areas the strongest LFP gamma-power was found in the superficial layers, gamma-coherence was stronger between V1-L2/3 and V2-L4 than between V1-L2/3 and V2-L2/3 (Figure 8E). Other studies examining V1-V4 interactions without precise laminar identification observed LFP-LFP gamma coherence values of about 0.1 (Bosman et al., 2012; Grothe et al., 2012). These LFP-LFP coherence values are difficult to interpret in terms of spiking output correlations because the LFP pools synaptic currents from multiple layers that derive both from local and distal spiking (Schomburg et al., 2014; Buzsáki and Schomburg, 2015). Grothe et al. (2012) quantified the SFC of superficial layer V4 cells to V1 gamma LFPs, and found that superficial-layer V4 spikes were only weakly entrained to V1 LFPs (SFC values of ~0.001). This weak V4-spikes-to-V1-LFP SFC stands in sharp contrast to the SFC values between L2/3 V4 cells and L2/3 V4 LFPs observed by previous studies (around 0.1; Fries et al., 2008; Buffalo et al., 2011).

Together, these data indicate that V1 gamma may not effectively propagate towards superficial V4 layers, and suggest that extrastriate areas can independently generate a superficial-layer gamma rhythm based on the integration of RF inputs with the surround (Figure 8A). The extent to which the upstream L2/3 gamma propagates towards the downstream area likely depends on both stimulus properties and behavioral/attentional state. Selective attention leads to a strong increase in gamma-coherence between areas V1 and V4 (Bosman et al., 2012; Grothe et al., 2012). Further, gamma-synchronization in the upstream area might entrain cells in the downstream area more effectively if the stimulus does not induce strong intrinsic gamma-synchronization in the downstream area. In anesthetized cats that have sufficient cortical activation (Herculano-Houzel et al., 1999), moving stimuli induce V1 gamma at a much lower frequency (~30–40 Hz) than in the LGN and the retina (~100 Hz), and for these stimuli the LGN gamma does not effectively entrain spiking in area V1 (Neuenschwander and Singer, 1996; Castelo-Branco et al., 1998). However, for stationary stimuli, V1 gamma is strongly reduced in anesthetized cats, with only few cells being entrained in the classic 30–40 Hz band (Gray et al., 1990). In this case, Castelo-Branco et al. (1998) found robust gamma-synchronization between the LGN and V1 in the 90–100 Hz band. The temporal structure of interareal correlations indicated a “synfire chain” (Abeles, 1982) communication pattern from retinal ganglion cells to LGN to V1 (Castelo-Branco et al., 1998).

Overall, these findings suggest the existence of a superficial-layer and a granular-layer gamma in visual cortex. The granular-layer gamma arises from entrainment by the upstream area and is gated by the superficial-layer gamma. While this communication scheme appears paradoxical from the point of view of feedforward information propagation, we note that there are fewer feedforward excitatory synapses from L4 to L2/3 in comparison to the total amount of recurrent L2/3 to L2/3 excitatory cortical synapses (Binzegger et al., 2004). In addition, studies from rodent indicate that excitatory L4-to-L2/3 synapses are on average not stronger than excitatory L2/3 to L2/3 synapses (Brecht, 2007).

## Gamma-Synchronization and Firing Rate Coding

We now shift attention towards the functional consequences of gamma-synchronization. An important realization is that gamma-synchronization does not occur in a computational void, but that its function must be understood in the context of information-rich firing rate representations: cortical computation cannot be understood without the consideration of cortical dynamics, and cortical dynamics cannot be understood without the consideration of cortical computation. Thus we first ask how gamma-synchronization relates to the information transmitted by V1 firing rates, and consider in particular the phenomenon of orientation tuning. The output of V1 cells can be interpreted as a stimulus likelihood function, with the likelihood landscape being sharper when the cell has a sharper orientation tuning curve (Jazayeri and Movshon, 2006). Incorporating predictions from the surround is expected to have important consequences for V1 firing rate representations. In particular, surround predictions should cause an improvement in the statistical inference of the CRF stimulus by V1 neurons, since additional evidence with independent noise can be taken into account. This follows from two known information-theoretic relationships. First, the remaining uncertainty about the CRF stimulus, quantified in number of bits, is reduced by inclusion of the surround data, i.e., H(S_CRF_|X_Surround_, X_CRF_) ≤ H(S_CRF_|X_CRF_) where S_CRF_ is the to-be-estimated stimulus in the CRF, and X_Surround_ and X_CRF_ are the input data that V1 receives. Second, we consider the Fisher information *I_x_(y)* (information in *x* about *y*), which is the expected curvature of the likelihood function around the maximum and gives a lower (Cramer-Rao) bound on the estimation error as Var(S_CRF_) = 1/I. Fisher information is additive, i.e., I_CRF_, _Surround_(S_CRF_) = I_CRF_(S_CRF_) + I_Surround_(S_CRF_), assuming that the CRF and surround input data are contaminated by independent noise. This means that, on average, the likelihood gets sharper around the maximum, and that estimation error decreases.

**Figure 9:**
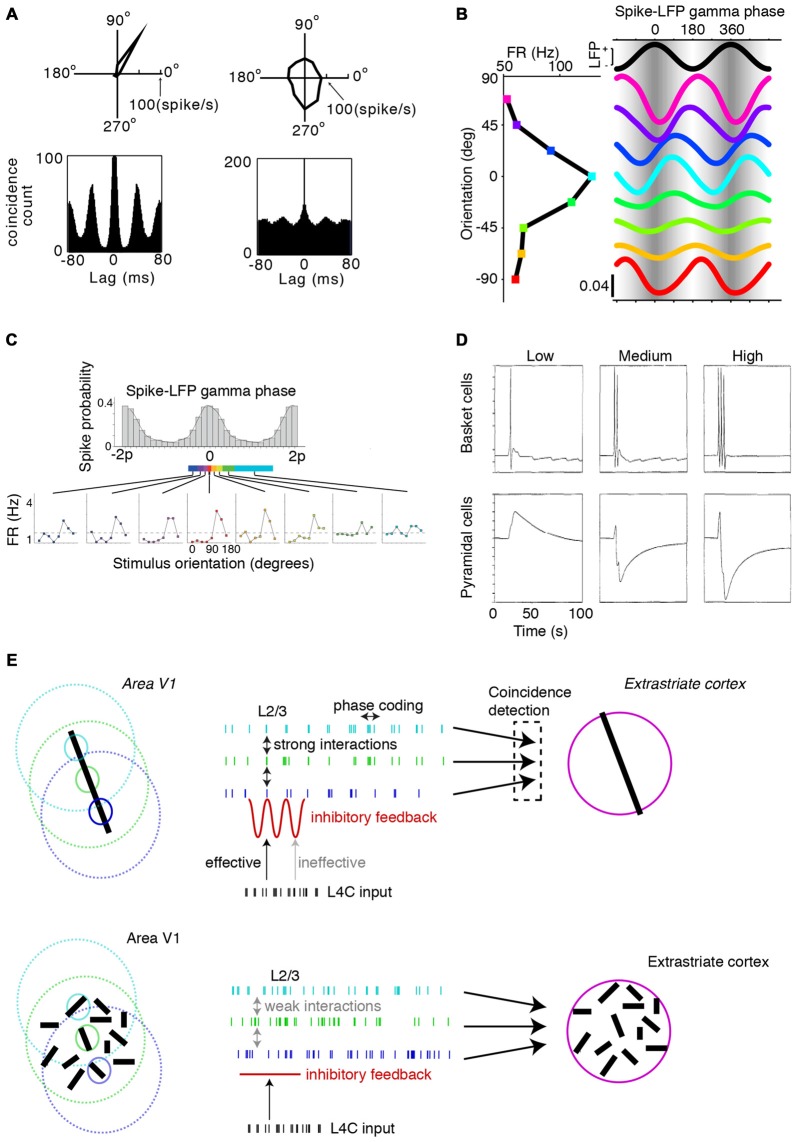
**Function of V1 gamma-synchronization. (A)** V1 recordings from superficial layers in anesthesized cat. Cells that show strong V1 gamma-synchronization are also sharply orientation tuned. Adapted from Folias et al. (2013). See also Womelsdorf et al. (2012) for a similar result obtained in the awake monkey. **(B)** Gamma spike phase code in area V1. Cells fire earlier in the gamma cycle when they are stimulated by a grating of their preferred orientation. Shown is a single unit’s average firing rate and spike phase density for eight different orientations. Adapted from Vinck et al. (2010). **(C)** V1 recordings were obtained from superficial layers in awake monkeys viewing a > 8° grating stimulus. The top panel shows the spike gamma phase histogram. For the bottom panel, gamma phase bins were determined such that each phase bin contained the same number of spikes. Virtual spike trains were then constructed by taking spikes only from one phase bin. Orientation tuning was then computed for each phase bin separately. The bottom panel shows that orientation tuning fluctuates gamma-rhythmically: the cell is more orientation tuned around the preferred gamma phase and less orientation tuned around the non-preferred gamma phase. Adapted from Womelsdorf et al. (2012). **(D)** In a network model, FS basket cells and pyramidal cells received excitatory currents. The FS basket cells provided feedforward inhibition onto the pyramidal cells. Small excitatory currents drive some spiking in FS basket cells and generate slowly decaying EPSPs (excitatory postsynaptic potentials) in pyramidal cells. Large excitatory currents give rise to strong feedback inhibition from FS basket cells, which can fire at very high rates. This gives rise to strong feedback inhibition in pyramidal cells and compresses the excitatory response in time, which can lead to a suppression of firing on average. Adapted from Bush and Sejnowski (1996). **(E)** Left, top: schematic illustrating functional consequences of gamma-synchronization. Top: under gamma-synchronized network dynamics, lateral, excitatory inputs are temporally convergent and escape feedback inhibition because they arrive at moments in time when GABAergic inhibition has waned off. These inputs therefore result in a strong drive, followed by strong feedback inhibition that makes the overall output sparse. The irregular inputs from L4C are therefore less effective over a large part of the gamma cycle in driving L2/3 cells, whereas the lateral inputs from L2/3 cells in other columns are more effective. Thus, gamma-synchronized network dynamics produce spiking output that is sparse, yet information-rich. Information content is further boosted through spike phase coding. Right, top: the synchronicity of the L2/3 output will, through feedforward coincidence detection, be more effective in triggering spikes in L4 cells of the next downstream area, which compensates for the loss of gain caused by the sparsification of spiking output. Left, bottom: irregular, asynchronous network dynamics are characterized by higher firing intensity in L2/3 cells, as well as inhibitory feedback that is more evenly spread in time. The lateral inputs from other columns are less effective in driving spiking, because inputs are not temporally convergent and do not arrive at a phase of weak GABAergic inhibition. Because spiking outputs are asynchronous, they are less effective in driving L4 cells of downstream areas than gamma-synchronous outputs.

The presence of a predictive surround in an image is indeed associated with sharper orientation tuning, higher feature selectivity in complex cells, and higher information rates per spike for natural scenes (Vinje and Gallant, 2000; Felsen et al., 2005; Okamoto et al., 2009; Pecka et al., 2014). Combined with the findings presented in Figures 1, 2, 3, 4, we conclude that gamma-synchronization tends to occur for stimuli for which response firing rates are particularly information-rich. This association appears to not only hold true across visual stimuli, but also across cells. In both anesthetized cats and awake monkeys there is a very strong linear association (Pearson’s R ~0.6–0.9) between gamma-synchronization and orientation tuning (Womelsdorf et al., 2012; Folias et al., 2013; Figure 9A). This finding suggests that cells that are part of the gamma-rhythmic network especially reap the benefits of CRF-surround integration on stimulus encoding.

## Functional Consequences of Gamma-Synchronization

### Properties of the Two Coding Modes

We have reviewed evidence demonstrating that, depending on the characteristics of the input image, visual cortex operates in a continuum of coding regimes that lie between two extremes. V1 spiking is irregular in the regime where firing outputs are predominantly driven by the CRF input, such that multiple columns with non-overlapping CRFs fire asynchronously (Figures 1, 2, 3, 4). On the other hand, the regime in which V1 firing rate outputs are influenced by the predictions from the surround has the following characteristics: (i) Spiking is strongly gamma-rhythmic (Figure 1, “The Relationship Between Gamma-Synchronization and Geometry” and “The Relationship Between Gamma-Synchronization and Motion Properties” Section). (ii) Coding is energy-efficient through the elimination of redundancies, reflecting the predictive component of surround suppression, as discussed in “Mechanisms of Gamma-Synchronization” Section. (iii) Firing rate variability is low (as measured by the Fano Factor; Figure 1B). (iv) Feature selectivity is high, as reviewed in “Relationship Between Gamma-Synchronization and Firing Rate Coding” Section. Together, these aspects indicate that in the gamma-rhythmic coding regime, neurons carry more information per spike about the CRF stimulus (Vinje and Gallant, 2000; Felsen et al., 2005; Okamoto et al., 2009).

### Functional Benefits of Gamma-Synchronization

Local gamma-synchronization could either play a functional role within a single visual area, or in the transmission of information from that visual area to downstream areas. These two possible roles are likely complementary, and we will discuss these separately in what follows.

#### Mechanistic Consequences of Gamma-Synchronization—Historical Overview

We first give a brief historical overview of previous proposals on the mechanistic role of gamma-synchronization. There have been four main influential ideas on the functional consequences of gamma-synchronization. For comprehensive reviews of these we refer to König et al. (1996), Singer et al. (1996), Salinas and Sejnowski (2001), Sejnowski and Paulsen (2006), Fries (2009, 2015), Vinck et al. (2013b) and Bosman et al. (2014).

(i) An influential idea is that gamma-synchronized spiking activity has an enhanced impact on post-synaptic targets as compared to asynchronous spiking activity (Abeles, 1982; Bernander et al., 1991; König et al., 1996; Kempter et al., 1998; Azouz and Gray, 2000; Salinas and Sejnowski, 2001; Fries, 2009; Vinck et al., 2013b). This increase in gain occurs through several mechanisms. A gamma-synchronized spiking output of the pre-synaptic population leads to an effective temporal summation of excitatory postsynaptic potentials in post-synaptic targets, thereby increasing the probability of reaching action potential threshold (Abeles, 1982; Bernander et al., 1991; König et al., 1996; Kempter et al., 1998; Azouz and Gray, 2000; Salinas and Sejnowski, 2001; Fries, 2009; Vinck et al., 2013b). This requires membrane time constants to be short. Effective integration times can be shortened when excitation is rapidly followed by feedback inhibition (Pouille and Scanziani, 2001). Also, there is evidence that neurons are particularly sensitive to inputs that are rapidly depolarizing (Azouz and Gray, 2000). Of special interest to this article is that synchrony can especially increase the gain in the sparse coding regime: First, a synchronous burst of spikes in the pre-synaptic population that causes the post-synaptic neuron to fire may contain more pre-synaptic spikes than needed to breach the action potential threshold (“overcrowding”). This surplus of spikes gets lost in the post-synaptic neuron’s refractory period. As a result, excessive synchrony can actually cause a decrease in gain (Bernander et al., 1991, 1994; Murthy and Fetz, 1994). Second, when the neurons in the post-synaptic population are in a relatively hyperpolarized state, small changes in the membrane potential variance induced by synchronization can cause large percent-wise changes in the probability of spike threshold crossing (Kempter et al., 1998). On the other hand, when the post-synaptic population is in a relatively depolarized state, an increase in synchrony only marginally or negatively affects the gain (Kempter et al., 1998).

(ii) Later work considered scenarios in which multiple neuronal groups have a gamma-rhythm, and examined how the integration of the synchronous input depends on the ongoing activity of the receiver (Fries, 2005, 2015; Börgers et al., 2008; Gielen et al., 2010; Akam and Kullmann, 2012). A core idea is that gain will be enhanced if excitatory input arrives at times of weak GABAergic inhibition, which can be achieved through phase-coupling of oscillations or entrainment (Azouz and Gray, 2003, 2008; Fries, 2005; Buzsáki, 2006; Womelsdorf et al., 2007).

(iii) Points (i) and (ii) focus on the transmission rather than the representation of information. It is a long-standing hypothesis that the brain uses the timing of spikes, in addition to the firing rate, to encode sensory information. One form of temporal coding is phase coding, in which the timing of spikes relative to an internal rhythm varies as a function of some variable (O’Keefe and Recce, 1993). Several studies have demonstrated that cells in V1 of cats and monkeys use a gamma phase code for stimulus orientation (Figure 9B; König et al., 1995; Maldonado et al., 2000; Fries et al., 2007; Tiesinga and Sejnowski, 2010; Vinck et al., 2010; Ballard and Jehee, 2011; Havenith et al., 2011). While phase coding could theoretically use any frequency, the visual system requires fast sensory readout and decoding times, for which the gamma-frequency band is more suited than lower frequency bands (Vinck et al., 2013b).

(iv) Finally, it has been pointed out that gamma-synchronization may regulate synaptic plasticity. Gamma-synchronization entails a precise organization of the timing of pre- and post-synaptic cells on the relevant time-scale of spike-time-dependent-plasticity (STDP; ~20 ms) and can therefore contribute to the induction of long-term synaptic potentiation or depression (Paulsen and Sejnowski, 2000; Buzsáki, 2006; Sejnowski and Paulsen, 2006; Vinck et al., 2010; Fell and Axmacher, 2011).

In what follows below, we will discuss the function of gamma-synchronization in the context of the proposed *Principles 1* and *2* and will incorporate these previous proposals about the mechanistic consequences of gamma-synchronization.

#### Local Functions of Gamma-Synchronization

We hypothesize that locally, gamma-synchronization solves a problem posed by the efficient/predictive coding scheme. The predictive coding scheme holds that information of the surround will be integrated with the CRF input such that the stimulus likelihood estimation performed by a column will be modified (“Relationship Between Gamma-Synchronization and Firing Rate Coding” Section). This leads to an average increase in stimulus information, as discussed in “Relationship Between Gamma-Synchronization and Firing Rate Coding” Section. Consequently, if the surround is predictive of CRF content, it needs to effectively drive spikes in excitatory cells (see “Excitatory Cell Responses” and “Network Dynamics” Sections). Yet, efficient and predictive coding schemes also indicate that firing become sparser if the surround is predictive of the CRF content (see “GABAergic Inhibition and Surround Suppression” Section). Thus, the surround influences should, paradoxically, both drive and sparsify the firing of L2/3 excitatory cells, under the constraints that synapses are on average weak and that the surround neurons fire sparsely themselves.

On the one hand, gamma-synchronization could play a role in suppressing the firing rate of L2/3 excitatory cells because the synchronous, convergent input from the surround triggers strong inhibitory feedback from local GABAergic interneurons (see “GABAergic Inhibition and Surround Suppression” Section; Figure 9D). This GABAergic inhibition remains strong for a large part of the gamma cycle and acts to decrease the firing rate of excitatory cells on average, leading to a sparser spiking output (Bush and Sejnowski, 1996; Tiesinga et al., 2004; Figures 9D,E). On the other hand, gamma-synchronization could enable the surround to actively contribute to spike generation in L2/3 excitatory cells. In each gamma cycle, there will be a synchronous, convergent excitatory input from the surround that arrives when the GABAergic inhibition wanes off (Figure 9E). Altogether, the temporal coincidence of synchronous excitatory inputs and low GABAergic inhibition should render the excitatory surround inputs particularly effective and lead to the generation of spikes in L2/3 excitatory cells (see “Mechanistic Consequences of Gamma-Synchronization – Historical Overview” Section). Through this mechanism, the surround can modify the stimulus likelihood representations for the CRF stimulus, thereby boosting the information content of spiking output (see “Relationship Between Gamma-Synchronization and Firing Rate Coding” Section). The efficiency of coding can be further enhanced by the utilization of phase coding, which can increase the amount of information transmitted per spike (see "Mechanistic Consequences of Gamma-Synchronization—Historical Overview" Section).

We expect that gamma-synchronized network dynamics entail an effective decoupling of L4C from L2/3 cells within the same column: recurrent L2/3 to L2/3 excitatory influences, which indirectly carry L4C inputs from other columns, should become more powerful whereas the direct influence of L4C over L2/3 firing within the same column will effectively be diminished (Figure 9E). This assertion is based on the idea that arrhythmic L4C inputs will be ineffective during a large part of the gamma cycle, because the synchronous drive from the surround triggers strong GABAergic inhibition (Figures 9D,E).

A prediction that follows is that orientation tuning itself fluctuates rhythmically over the gamma cycle, because the surround inputs will be largely concentrated in one phase of the gamma cycle, while the arrhythmic L4C inputs are scattered throughout the gamma cycle. In support, Womelsdorf et al. (2012) have shown that neurons tend to more orientation tuned around the peak of the gamma cycle, even after correcting for the number of spikes (Figure 9C).

#### Functions of Gamma-Synchronization in Interareal Communication

We have reviewed data suggesting that sparse coding is accompanied by gamma-synchronization. This might have an important function for the transmission of signals to other brain areas. A typical visual scene comprises multiple objects that compete for the processing resources of higher brain areas. Reynolds and Desimone (1999) have shown that when multiple objects are positioned within the RF of a neuron in extrastriate cortex, the response of this neuron is not the sum of the individual inputs, but rather a weighted average of the distinct inputs. Visual inputs that are not sparsely encoded, i.e., give rise to higher firing activity, could thus bias the competition in higher visual areas towards them and away from visual inputs that are sparsely encoded (Reynolds and Desimone, 2003). Further, the routing of sparse spiking output to other brain areas might be more vulnerable to transmission noise than the routing of non-sparse spiking output. Thus, the sparse encoding of information might have disadvantages in terms of information transmission to other brain areas. We hypothesize that gamma-synchronization provides an elegant solution to this problem by increasing the effective gain that sparse V1 activity has on post-synaptic targets in other brain areas.

### Functional Benefits of Irregular Firing

The question about the function of gamma-synchronization has been frequently asked (Fries, 2009; Ray and Maunsell, 2015), but we should likewise ask what the evolutionary benefit is of the irregular spiking output that can be found for certain stimuli (Figures 1, 2). We believe that these two questions are two sides of the same coin that beg to be considered in tandem. One possibility is that the irregular, asynchronous coding mode is a functional phenomenon that prevents interference of surround activity on the CRF representations when it does not provide information. That is, if the spike trains are temporally uncorrelated, then this might be the optimal coding regime for a stimulus where the coding at each retinotopic location needs to be independent (e.g., white noise or small stimulus): If multiple neuronal populations in the surround fire asynchronously, then we expect their spiking outputs to be less effective in driving the L2/3 output of the CRF column. Further, asynchronous, irregular firing might prevent STDP to occur (Sejnowski and Paulsen, 2006). Thus, we conjecture that the irregularity of spiking output is a functional characteristic that prevents interference from the surround inputs for specific stimuli, rather than a generic property of neural firing. It is well established that in this coding regime, the visual cortex can encode a substantial amount of information using the firing rate, although it remains controversial whether this provides a sufficiently large channel capacity to encode the visual input in small time intervals (Gautrais and Thorpe, 1998). An alternative interpretation is that neuronal output appears Poisson-like when spike train statistics are quantified with conventional means, but that information is still encoded through sequences of action potentials in which the relative latency of spiking between cells signals information (Ballard and Jehee, 2011).

## Conclusion

In sum, in this article we have reviewed evidence showing that gamma-synchronization arises in very specific circumstances that require predictive integration of CRF and surround data, whereas irregular, asynchronous firing arises when no CRF-surround integration takes place and interference has to be avoided. The resulting gamma-synchronous dynamics that depend on extensive, recurrent superficial patchy networks are likely critical for the emergence of sparse and highly informative firing rate representations that are effectively routed to the next brain area.

## Author Contributions

MV drafted manuscript, in discussion with CAB; CAB edited the manuscript.

## Conflict of Interest Statement

The authors declare that the research was conducted in the absence of any commercial or financial relationships that could be construed as a potential conflict of interest.

## Appendix

Here we discuss problems with the comparison of SFC between areas in Buffalo et al. (2011; see “Gamma Across the Visual Hierarchy” Section). An additional goal is to illustrate some factors that need to be considered in order to compare SFC values between areas. We emphasize that the Buffalo et al. (2011) study was not designed to compare the strength of gamma-synchronization between different visual areas, but rather to investigate laminar differences in SFC and its attentional modulation (see “Laminar Dependence of Gamma-Synchronization” Section). Our concerns arise from an attempt to use this data for other purposes, and do not undermine the conclusions drawn by Buffalo et al. (2011) about laminar differences in SFC and its attentional modulation. Furthermore, these considerations may also apply to some other studies reviewed in “Gamma Across the Visual Hierarchy” Section (e.g., Chalk et al., 2010).

(i) In Buffalo et al. (2011), the superficial V2 data contained only 13 units (vs. 67 in V1). The authors write that “Spikes were sorted into single units. When this was not possible, multiunits were accepted.” Hence, the V2 data comprised only a few sessions (<7) because multiple electrodes in superficial layers were used (2–4), such that the effective number of independent data points (and the statistical power) was quite small. Judging from the n’s, the V2 data likely derives primarily or exclusively from one monkey. (The authors write that “In some layers and areas, we obtained data primarily from one or the other monkey”). The data from e.g., superficial V1 and V4 stem from two monkeys (containing a different and unknown mixture of units across monkeys). Hence, it is difficult if not impossible to make a valid comparison of SFC values between areas given the known, large systematic variability in gamma-synchronization across subjects (van Pelt et al., 2012).

(ii) The analysis in Buffalo et al. (2011) is based on SFC, which has been the standard measure of spike-field correlations in the field for many years. The SFC has a strong, positive dependence on the firing rate, which is especially problematic when using multi-units (Zeitler et al., 2006; Vinck et al., 2012; Lepage et al., 2013). Without further controls, this bias makes it difficult to compare SFC values between areas. Because of the low number of units (and sessions) for area V2 (*n* = 13), it is also possible that the V2 data contained more multi-units than V1 (*n* = 67) and V4 (*n* = 73). Further, the authors of Buffalo et al. (2011) do not report the MUA firing rate, but judging from Figure 1 in Fries et al. (2001), whose V4 multi-units were included in Buffalo et al. (2011), the thresholds used for the V4 dataset gave rise to quite high firing rates (around 100 Hz). This means that one can strongly inflate SFC values as compared to single unit data (for a direct comparison, see Zeitler et al., 2006). Depending on the online recording threshold, the MUA can have different firing rates across areas.

(iii) Buffalo et al. (2011) did not necessarily use the same stimulus eccentricity for all visual areas (this depended on the electrode penetration site), which makes a direct comparison between areas difficult because we do not know the RF sizes of V1, V2 and V4 sites.

(iv) Figure S1 in Buffalo et al. (2011) shows much less variability in SFC values in area V2 than in area V1. In fact, many V1 sites are unlikely to even have a gamma-band response, given that the SFC values are around the random level (see their Figure 1), with about half of the V1 sites have SFC values <0.1. It is unclear why the V1 SFC was much more variable than the V2 SFC. This might result from sampling of particular cortical layers, the use of various eccentricities, the inclusion of more single units or other factors. In Buffalo et al. (2011), electrodes were lowered as needed to increase the neuronal signal, and layers were identified based on recording depth, with a maximum recording depth of 1 mm for superficial layers in area V1. This can put the electrodes well in the granular layers given that the cortical thickness of area V1 in macaque is about 1.5–2 mm (e.g., see Lund et al., 2003). Because V2 is approached from the deep layers, there might be systematic differences in layers with V1 recordings. This issue might be especially important given that V1 gamma-synchronization tends to be weak in the middle layer (see “Relative Importance of Lateral and Extrastriate Feedback Connections.” Section), while V2 and V4 gamma-synchronization tend to be stronger in the middle layers because of entrainment by V1 activity (see “Gamma Across the Visual Hierarchy” Section).

As a practical advice, in order to compare gamma-synchronization between areas, it is important to use metrics that are not biased by firing rate, as well as controlling the cortical layer and the RF size of cells (which in each area depends positively on eccentricity). Ideally, this is achieved using simultaneous recordings, which also removes variance from behavior.
